# Matrine Suppresses Osteosarcoma Lung Metastasis by Targeting the PI3K/AKT Signaling Pathway to Induce Mitochondrial Dysfunction

**DOI:** 10.3390/cancers18203381

**Published:** 2026-10-19

**Authors:** Chanyi Jiang, Xiankun Liu, Yijun Yu, Hongming Lin, Xiaochun Xiong, Yinghao Yu, Songou Zhang

**Affiliations:** 1 Department of Pharmacy, Zhoushan Hospital of Traditional Chinese Medicine Affiliated to Zhejiang Chinese Medical University, Zhoushan 316000, China; jcy88102730@126.com; 2 Department of Orthopedics, Zhoushan Hospital of Traditional Chinese Medicine Affiliated to Zhejiang Chinese Medical University, Zhoushan 316000, China; xkunliu@163.com (X.L.);; 3 Medical Research Center, Zhoushan Hospital of Traditional Chinese Medicine Affiliated to Zhejiang Chinese Medical University, Zhoushan 316000, China; 4 School of Medicine, Ningbo University, Ningbo 315000, China; 5 Department of Orthopedics, Shaoxing People’s Hospital, No. 123 Baiyutan Road, Yuecheng District, Shaoxing 312000, China

**Keywords:** matrine, osteosarcoma, lung metastasis, PI3K/AKT signaling pathway, mitochondrial dysfunction, apoptosis

## Abstract

**Objective**: Osteosarcoma, the most common primary bone malignancy in children and adolescents, frequently metastasizes to the lung, with a five-year survival rate of about 20% after lung metastasis. This study assessed whether matrine suppresses osteosarcoma lung metastasis by inducing mitochondrial dysfunction through inhibition of the PI3K/AKT pathway. **Methods**: Using 143B, U2OS, and MG63 cells, we assessed proliferation, colony formation, and invasion; examined mitochondrial ultrastructure, membrane potential, and reactive oxygen species (ROS); and measured PI3K/AKT phosphorylation and mitochondrial dynamics and apoptosis-related proteins by Western blotting. Direct binding of matrine to PI3K was verified by molecular docking and surface plasmon resonance (SPR). Rescue experiments used the PI3K agonist 740 Y-P, and efficacy was tested in subcutaneous xenograft and tail vein lung metastasis models. **Results**: Matrine inhibited the proliferation, colony formation, and invasion of 143B, U2OS, and MG63 cells in a concentration-dependent manner, with 143B cells being the most sensitive, and in vivo, it suppressed tumor growth and lung metastasis without obvious organ toxicity. Mechanistically, matrine caused mitochondrial swelling, cristae rupture, membrane potential loss, and ROS accumulation; upregulated FIS1, Bax, and cytochrome c; and downregulated Mfn1 and Mfn2. It directly bound to PI3K and inhibited PI3K/AKT phosphorylation. The PI3K agonist 740 Y-P only partially reversed these effects, indicating partial dependence on the PI3K/AKT pathway. **Conclusions**: Matrine suppresses osteosarcoma lung metastasis by targeting the PI3K/AKT pathway to induce mitochondrial dysfunction and may be a useful therapeutic agent.

## Simple Summary

Osteosarcoma survival drops to ~20% after lung metastasis, highlighting the need for new therapies. This study shows that matrine, a natural compound from *Sophora flavescens*, suppresses osteosarcoma growth and lung metastasis in vitro and in vivo by directly inhibiting PI3K/AKT signaling, thereby inducing mitochondrial dysfunction, fission, and apoptosis. Matrine represents a promising therapeutic candidate for osteosarcoma, particularly for preventing lethal lung metastasis.

## 1. Introduction

Osteosarcoma is the most common primary malignant bone tumor in children and adolescents. Its biological behavior is highly aggressive, and it tends to metastasize to the lung through the bloodstream at an early stage [1,2,3,4]. Clinical data show that the five-year survival rate of patients with localized osteosarcoma can reach 65% to 75% after neoadjuvant chemotherapy combined with surgical resection. However, once lung metastasis occurs, the five-year survival rate drops to below 20%, with no significant improvement over the past three decades [1]. The survival benefit of resection of lung metastatic lesions remains controversial, and current chemotherapeutic drugs face efficacy bottlenecks and dose-limiting toxicity [5,6,7]. Therefore, there is an urgent need for novel antimetastatic drugs that are highly effective and less toxic, especially those targeting the cascade of lung metastasis.

Natural products occupy an important position in anticancer drug development because of their multiple targets and low toxicity [8,9]. Matrine, a quinolizidine alkaloid extracted from the leguminous plant Sophora flavescens, has drawn increasing interest in tumor pharmacology [10,11]. Matrine exerts anticancer effects through multiple pathways. In cervical cancer, it activates the Piezo1 channel to induce ferroptosis [12]. In breast cancer, it regulates the LINC01116/miR-9-5p/ITGB1 axis to suppress epithelial–mesenchymal transition (EMT) [13]. In liver cancer and colorectal cancer, it remodels metabolism and alleviates oxidative stress through noncoding RNA networks [14,15]. These studies indicate that matrine can restrain tumor progression through mechanisms that include ferroptosis induction, apoptosis regulation, and metabolic reprogramming [16]. Molecularly targeted therapies have emerged as more and more significant in cancer research, with special emphasis on the identification of key regulators of tumor cell survival, proliferation, and apoptosis. Recent evidence has suggested that targeting critical signaling and regulatory pathways, such as the MDM2/p53 axis, may have therapeutic relevance in regulating tumor progression and treatment response. The results here further support the investigation of natural products that can affect multiple molecular pathways associated with a malignancy, including matrine, in the more aggressive forms of the disease [17]. However, compared with other solid tumors, studies on matrine in osteosarcoma remain very limited. Matrine has also been shown to inhibit osteosarcoma cell proliferation through the mitogen-activated protein kinase/extracellular signal-regulated kinase (MAPK/ERK) pathway [18]. However, that work was limited to the proliferative phenotype and did not address lung metastasis or mitochondrial mechanisms. Systematic experimental evidence on the effect of matrine against osteosarcoma lung metastasis, a fatal process, and its molecular mechanisms is still lacking.

The phosphatidylinositol 3-kinase/protein kinase B (PI3K/AKT) signaling pathway not only drives the proliferation and metastasis of osteosarcoma but also participates in the regulation of mitochondrial dynamics in multiple tumors [19,20]. We therefore proposed the hypothesis that matrine may suppress osteosarcoma lung metastasis by inhibiting the PI3K/AKT signaling pathway and disturbing mitochondrial dynamics. To test this hypothesis, we combined network pharmacology prediction with molecular docking, SPR, and the cellular thermal shift assay (CETSA) to confirm the direct binding of matrine to PI3K at the molecular level. We then systematically evaluated the antimetastatic efficacy of matrine in vitro and in vivo using a tail vein lung metastasis model. Transmission electron microscopy, flow cytometry, and Western blotting were used to reveal mitochondrial morphological and functional damage and the activation of mitochondria-dependent apoptosis. Finally, a rescue experiment with the PI3K agonist 740 Y-P was performed to establish the causal role of the PI3K/AKT pathway. This study sought to define the molecular mechanism by which matrine suppresses osteosarcoma lung metastasis and to provide experimental support for natural-product-based treatment of osteosarcoma.

## 2. Methods

### 2.1. Cell Culture

Human osteosarcoma cell lines 143B, U2OS, and MG63 were purchased from Procell Life Science & Technology Co., Ltd. (Wuhan, China) and cultured in DMEM supplemented with 10% FBS and 1% penicillin–streptomycin at 37 °C with 5% CO_2_.

### 2.2. CCK-8 Assay

The Cell Counting Kit-8 (CCK-8, Beyotime) assay was used to examine the effect of matrine on the proliferation of 143B, U2OS, and MG63 cells. Logarithmically growing 143B cells were seeded into 96 well plates at 3 × 10^3^ cells per well in 100 μL of medium. After 24 h of culture, the old medium was removed and replaced with fresh medium containing matrine at different concentrations. After another 24 h, 10 μL of CCK-8 solution was added to each well and incubated in the dark for 2 h. The absorbance at 450 nm was measured with a microplate reader (BioTek, Winooski, VT, USA). Cell viability was calculated, growth curves were plotted, and the half maximal inhibitory concentration (IC_50_) was calculated using GraphPad Prism 8.0 software.

### 2.3. Colony Formation Assay

To evaluate the effect of matrine on long-term proliferative potential, a colony formation assay was performed. 143B, U2OS, and MG63 cells were seeded into 6 well plates at 500 cells per well. After 24 h, the medium was replaced with medium containing matrine or the agonist at different concentrations. The drug containing medium was changed every 3 days, and the cells were cultured for 14 days. Culture was terminated when colonies became visible to the naked eye. Cells were fixed with 4% paraformaldehyde and stained with crystal violet. Excess dye was removed by gentle rinsing with running water. The plates were dried at room temperature and photographed, and the colony formation rate was calculated.

### 2.4. Invasion Assay

The Transwell assay was used to assess the effect of matrine on the invasive ability of 143B, U2OS, and MG63 cells. The upper chambers were precoated with Matrigel, and cells (2 × 10^5^ cells/mL, 200 μL) were seeded into the upper chamber in serum-free medium, with medium containing 20% FBS added to the lower chamber as a chemoattractant. Different concentrations of matrine or the agonist were added to the upper chamber. After 24 h, non-invaded cells were removed, and cells on the lower membrane surface were fixed and stained with crystal violet for visualization and counting.

### 2.5. In Vivo Tumor Xenograft Experiment

All animal experiments were approved by the institutional animal ethics committee and followed the 3R principles. Female BALB/c nude mice (4–6 weeks old, SPF grade) were purchased from Shanghai SLAC Laboratory Animal Co., Ltd. (Shanghai, China). 143B cells (1 × 10^6^ cells in 0.1 mL PBS) were injected subcutaneously. Tumor-bearing mice were randomly divided into five groups (n = 6 per group): control (normal saline), cisplatin (10 mg/kg), low-dose matrine (5 mg/kg), medium-dose matrine (20 mg/kg), and high-dose matrine (40 mg/kg). Drugs were administered intraperitoneally once daily for 21 consecutive days. At the endpoint, tumors were imaged via an in vivo imaging system after D-luciferin injection, and the mice were euthanized; tumors were then dissected, weighed, and photographed.

### 2.6. Lung Metastasis Model

To evaluate the inhibitory effect of matrine on osteosarcoma lung metastasis, a tail vein metastasis model was established by injecting 143B cells (5 × 10^5^ cells in 0.1 mL PBS) into BALB/c nude mice. Drug administration began the next day. Mice were divided into the same five groups as described in Section 2.5. Body weight and general condition were monitored regularly. At the endpoint, lung metastasis was assessed using an in vivo imaging system, and lung tissues were collected for subsequent analysis.

### 2.7. Hematoxylin and Eosin (H&E) Staining

Lung tissues were fixed in 4% paraformaldehyde for 24 h, dehydrated through a graded ethanol series, cleared in xylene, and embedded in paraffin. Serial 4-μm sections were prepared and stained with H&E. Pathological changes in the lung tissue, including the number, size, and infiltration of tumor metastatic nodules, were observed and recorded under a light microscope.

### 2.8. Transmission Electron Microscopy Observation of Mitochondrial Ultrastructure

Transmission electron microscopy was used to examine the effect of matrine on mitochondrial ultrastructure in 143B cells. Cells were treated with matrine (0, 0.5, 1, and 2 μM) for 24 h, then fixed in 2.5% glutaraldehyde, postfixed with 1% osmium tetroxide, and embedded in epoxy resin. Ultrathin sections were double-stained and observed under a transmission electron microscope.

### 2.9. JC-1 Flow Cytometry for Mitochondrial Membrane Potential

JC-1 fluorescent staining combined with flow cytometry was used to evaluate the effect of matrine on the mitochondrial membrane potential. 143B cells were treated with matrine at different concentrations (0, 0.5, 1, and 2 μM) for 24 h. The cells were collected and incubated with JC-1 staining working solution (Beyotime, Shanghai, China) at 37 °C in the dark for 20 min. After washing with JC-1 staining buffer, the fluorescence signals were detected by flow cytometry. The ratio of red fluorescence (JC-1 aggregates) to green fluorescence (JC-1 monomers) reflected the change in mitochondrial membrane potential.

### 2.10. Flow Cytometry for Intracellular ROS Levels

Intracellular ROS levels were measured using the DCFH-DA fluorescent probe. 143B cells were treated with matrine as described in Section 2.8, and then incubated with DCFH-DA (10 μM) at 37 °C in the dark for 30 min. After washing, DCF fluorescence was detected by flow cytometry, and the mean fluorescence intensity (MFI) was used to reflect intracellular ROS levels.

### 2.11. Western Blotting for Protein Expression

Western blotting was used to detect the expression of mitochondrial dynamics and apoptosis-related proteins. 143B cells were treated with matrine as described in Section 2.8, and then collected. Total protein was extracted by lysing the cells on ice for 30 min with radioimmunoprecipitation assay (RIPA) lysis buffer (Beyotime, Shanghai, China) containing protease inhibitors. After centrifugation, the supernatant was collected, and the protein concentration was determined with a bicinchoninic acid (BCA) protein assay kit (Beyotime, Shanghai, China). Equal amounts of protein were separated by SDS-PAGE, transferred to PVDF membranes, and blocked with 5% skim milk for 1 h. Membranes were incubated overnight at 4 °C with primary antibodies against Mfn1, Mfn2, FIS1, Bax, cytochrome c, and β-actin, followed by incubation with HRP-conjugated secondary antibodies for 1 h at room temperature. Antibodies were obtained from Cell Signaling Technology (CST), Proteintech, and Diagbio. Specific catalog numbers and dilution ratios can be found in Table S1. Signals were detected using ECL reagent (Beyotime, Shanghai, China), and band intensities were quantified with ImageJ V1.48 using β-actin as the internal control. The original blot can be found in Supplementary File S1.

### 2.12. Rescue Experiment with the PI3K Agonist

To verify whether the effect of matrine depends on the PI3K/AKT signaling pathway, a rescue experiment was performed with the PI3K agonist 740 Y-P. 740 Y-P is a cell-permeable phosphopeptide that specifically binds to the p85 regulatory subunit of PI3K and activates the PI3K/AKT signaling pathway. The working concentration (10 μM) was selected based on a pilot dose–response assay and is consistent with concentrations reported to effectively activate PI3K/AKT signaling in previous studies [21]. 740 Y-P was dissolved in sterile distilled water and added to the culture medium; the medium was replaced every 24 h to maintain stable pathway activation. In vitro, 143B cells were divided into four groups: control, 740 Y-P alone, matrine alone, and matrine + 740 Y-P. Cells were pretreated with 740 Y-P for 1 h before matrine administration, after which the two agents were co-administered for the duration of each assay. In vivo, mice in the lung metastasis model received intraperitoneal injection of 740 Y-P (10 mg/kg) in addition to matrine. 740 Y-P was administered 1 h before each matrine dose, once daily for 21 consecutive days, with the dosing schedule aligned with that of matrine. The 10 mg/kg dose was selected based on previous in vivo studies demonstrating effective PI3K/AKT activation without overt toxicity [22].

### 2.13. Screening of Drug and Disease Targets and Construction of the PPI Network

To systematically identify the potential targets of matrine against osteosarcoma metastasis, a strategy integrating multiple databases was adopted. All databases were accessed in October 2025, and the retrieved records were archived to ensure reproducibility.

Putative targets of matrine were retrieved from the Traditional Chinese Medicine Systems Pharmacology Database (TCMSP), SwissTargetPrediction, PharmMapper, and BindingDB. Targets were queried using the canonical structure of matrine. For SwissTargetPrediction, only predictions with a probability ≥0.5 were retained; for PharmMapper, only hits with a normalized fit score ≥0.5 were retained; for TCMSP, targets associated with matrine (MOL001924) were collected directly. Duplicate entries and invalid records were removed, and target names were standardized to official gene symbols using the UniProt database. Osteosarcoma-related genes were retrieved from GeneCards, Online Mendelian Inheritance in Man (OMIM), DisGeNET, and the Therapeutic Target Database (TTD) using the keyword “osteosarcoma.” From GeneCards, genes with a relevance score ≥10 were retained, and genes with lower scores were excluded as annotations of limited specificity. Duplicates were removed and genes not causally related to osteosarcoma were excluded by manual curation. The drug-target set and disease-target set were intersected using the VennDiagram package in R to obtain the common targets, which constituted the core target group of matrine against osteosarcoma metastasis. These core targets were imported into the STRING database (confidence score ≥ 0.7, species limited to *Homo sapiens*, disconnected nodes hidden) to construct a protein–protein interaction (PPI) network, which was visualized with Cytoscape (v3.9.1). Topological parameters including degree centrality and betweenness centrality were calculated with the NetworkAnalyzer plugin. Hub genes were defined as those with a degree value ≥twice the median degree of the network, and the top-ranked genes were selected to construct the core regulatory network. GO (Biological Process, Cellular Component, Molecular Function) and KEGG pathway enrichment analyses were performed on the core targets using the clusterProfiler package in R. Enrichment was considered statistically significant only after adjustment for multiple testing using the Benjamini–Hochberg (false discovery rate, FDR) method, with an adjusted *p* value (q < 0.05) as the threshold; the nominal *p* < 0.05 threshold alone was not used as the sole criterion for pathway significance. The analysis output included the term description, enriched gene count, fold enrichment, raw *p* value, adjusted *p* value, and gene ratio for each term.

### 2.14. Functional and Pathway Enrichment Analyses of Key Targets

To further clarify the biological functions of the core targets, Gene Ontology (GO) functional enrichment and Kyoto Encyclopedia of Genes and Genomes (KEGG) pathway enrichment analyses were performed on the screened hub genes using the DAVID database. The GO analysis covered three dimensions: biological process (BP), molecular function (MF), and cellular component (CC), with emphasis on cell migration, apoptosis regulation, and mitochondrial function. The KEGG analysis focused on signaling pathways closely related to tumor metastasis. A significance threshold of *p* < 0.05 was used. The enrichment results were visualized with bubble plots and chord diagrams.

### 2.15. Mitochondrial Respiration Assay

Mitochondrial respiratory function was assessed using a Seahorse XF Cell Mito Stress Test Kit according to the manufacturer’s instructions. 143B cells were seeded in XF 96-well microplates and treated with matrine alone or in combination with the PI3K agonist 740 Y-P. Prior to the assay, cells were incubated in XF Base Medium supplemented with glucose, pyruvate, and glutamine for 1 h. Oxygen consumption rate (OCR) was measured under basal conditions and following sequential injection of oligomycin, FCCP, and rotenone/antimycin A. Data were normalized to cell number.

### 2.16. Statistical Analysis

Statistical analyses and graphing were performed using GraphPad Prism 8.0. Data are presented as the mean ± SD from at least three independent biological experiments, unless otherwise indicated. The number of biological replicates (n) is specified in each figure legend. Normality and homogeneity of variance were assessed using the Shapiro–Wilk and Levene’s tests, respectively. Two-group comparisons used Student’s unpaired two-tailed *t* test; multigroup comparisons used one-way ANOVA followed by Tukey’s post hoc test. *p* values were adjusted for multiple comparisons using the Benjamini–Hochberg FDR method, with statistical significance defined as adjusted *p* < 0.05. For body weight and other variables measured repeatedly over time, two-way repeated-measures ANOVA was used to assess the effects of treatment, time, and their interaction.

## 3. Results

### 3.1. Matrine Inhibits the Proliferation, Colony Formation, and Invasion of Human Osteosarcoma Cells In Vitro

**Figure 1:**
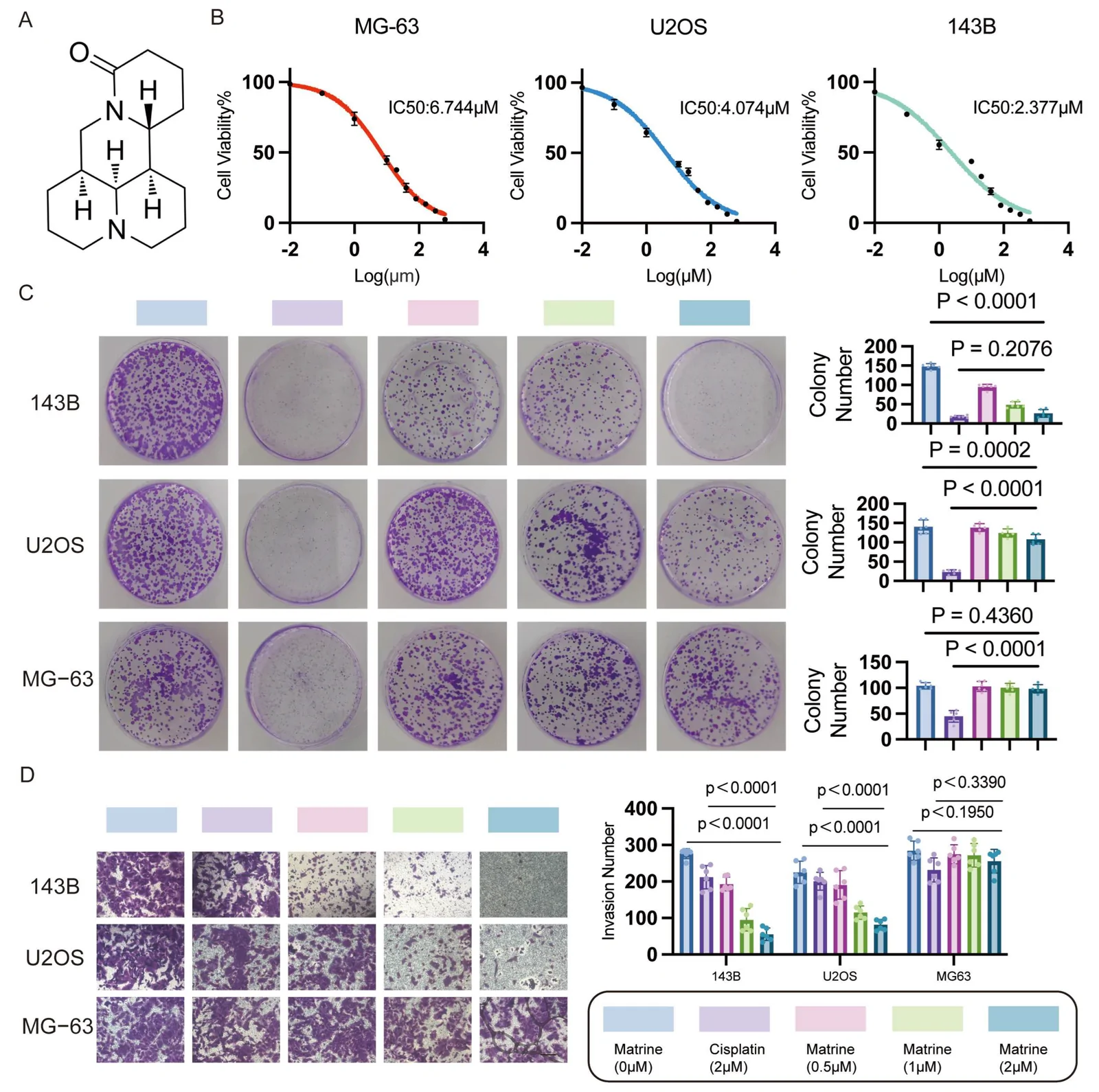
Matrine inhibits the proliferation, colony formation, and invasion of human osteosarcoma cells in vitro. (**A**) Chemical structure of matrine. (**B**) CCK-8 assay of 143B, U2OS, and MG63 cells treated with increasing concentrations of matrine for 24 h. (**C**) Colony formation assay of the three cell lines treated with matrine or cisplatin (10 μM). (**D**) Transwell invasion assay of the three cell lines treated with matrine or cisplatin (10 μM). Scale bar, 50 μm. Data are presented as the mean ± SD. *n* = 6.

To evaluate the in vitro antitumor activity of matrine against human osteosarcoma cells, three osteosarcoma cell lines (143B, U2OS, and MG63) were used, with cisplatin (10 μM), a first-line chemotherapeutic drug, as the positive control. The effects of matrine on cell viability, colony formation, and invasion were examined. Figure 1A shows the molecular structure of matrine. In the CCK-8 assay, matrine inhibited the proliferation of the three cell lines in a concentration-dependent manner, with IC_50_ values of 2.377, 4.074, and 6.744 μM, respectively. The 143B cells were the most sensitive (Figure 1B). In the colony formation assay, the number of colonies in all three cell lines decreased as the matrine concentration increased. In 143B cells, the mean colony number decreased from 148.2 ± 6.9 to 26.5 ± 10.0 at 2 μM (*p* < 0.0001). The colony number in U2OS cells also decreased significantly, whereas MG63 cells were largely unaffected. The inhibition of colony formation was more pronounced in the cisplatin group (Figure 1C). The Transwell invasion assay showed that matrine reduced the number of invading cells in a concentration-dependent manner, with the strongest effect on 143B cells. The number of invading cells decreased from 259.7 ± 30.6 to 68.5 ± 6.7 at 2 μM (*p* < 0.0001). The inhibitory effect of cisplatin on invasion was stronger than that of matrine (Figure 1D). hFOB 1.19 osteoblasts and BMSCs were included. Both cell types remained viable at ≤16 μM, whereas the osteosarcoma cells were markedly inhibited at 2–4 μM, suggesting that matrine may preferentially target osteosarcoma cells over non-malignant cells (Figure S1). Thus, matrine inhibited the proliferation, colony formation, and invasion of human osteosarcoma cells in vitro, albeit less potently than cisplatin.

### 3.2. Matrine Inhibits the Proliferation of 143B Human Osteosarcoma Cells In Vivo

**Figure 2:**
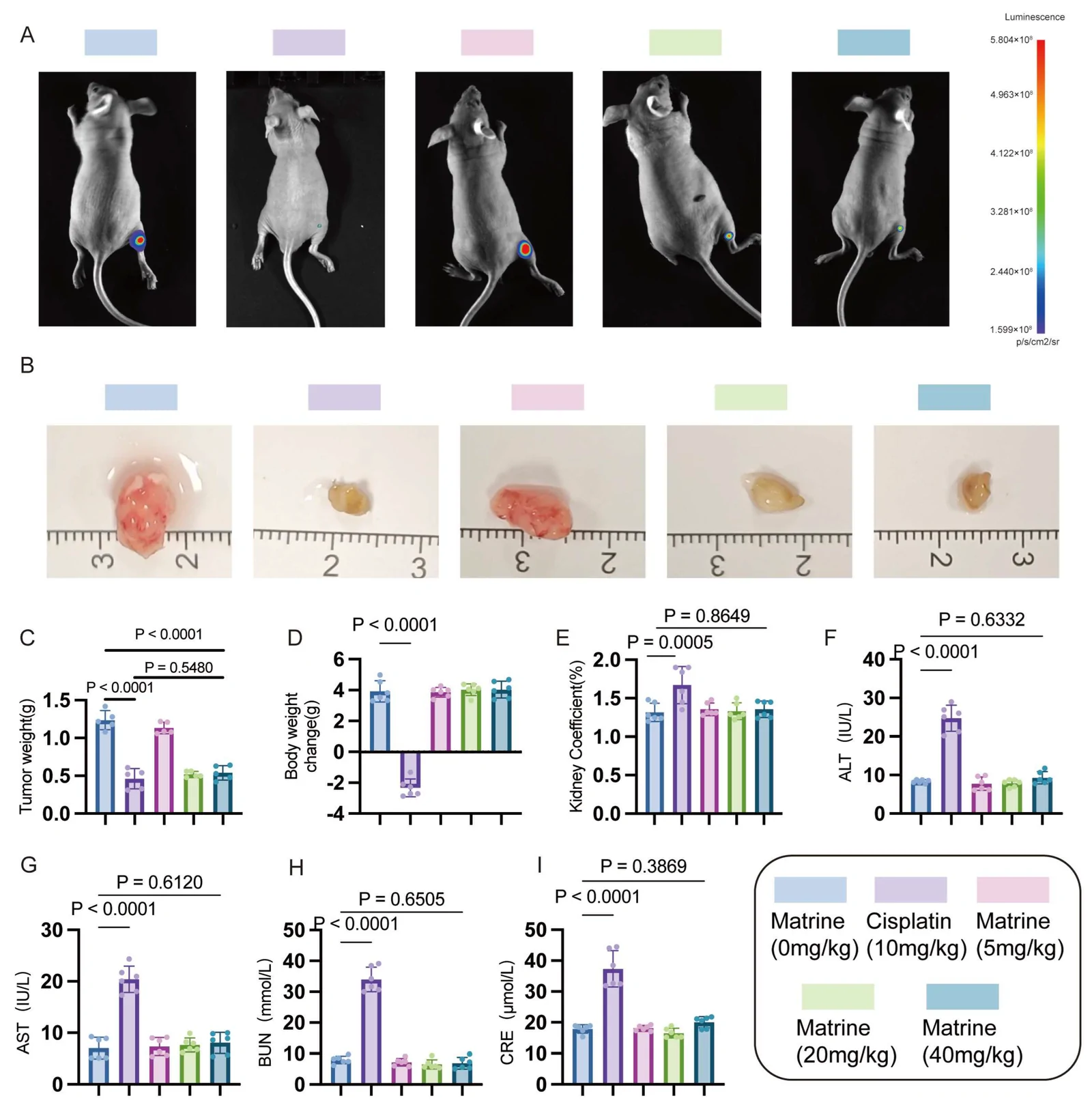
Matrine inhibits tumor growth in vivo without obvious toxicity. (**A**) In vivo bioluminescence imaging of tumor-bearing mice in the indicated groups. (**B**) Representative images of dissected tumors. (**C**) Tumor weight. (**D**) Body weight changes during treatment. (**E**) Kidney index. (**F**) Alanine aminotransferase (ALT). (**G**) Aspartate aminotransferase (AST). (**H**) Blood urea nitrogen (BUN). (**I**) Creatinine (CRE). Data are presented as the mean ± SD. *n* = 6.

To evaluate the in vivo antitumor effect and safety of matrine against osteosarcoma, a subcutaneous xenograft model of human osteosarcoma 143B cells was established in nude mice. The tumor-bearing mice were randomly divided into the control group, the cisplatin group (10 mg/kg), and the low-, medium-, and high-dose matrine groups (5, 20, and 40 mg/kg). Drugs were administered by intraperitoneal injection once daily for 21 consecutive days. In vivo imaging revealed that the bioluminescence signals of tumors in the cisplatin group and all matrine groups were significantly weaker than those in the control group, and the signals decreased as the matrine dose increased (Figure 2A). The gross appearance of the dissected tumors also showed that tumors in the control group were markedly larger and heavier than in all treatment groups (Figure 2B,C). This suggested that matrine inhibited osteosarcoma growth in vivo in a dose-dependent manner. Regarding safety, the body weight of control mice gradually increased during treatment, whereas that of cisplatin-treated mice decreased significantly (*p* < 0.0001). The body weight changes in the matrine groups showed no significant difference from the control group (Figure 2D). The serum and organ indices showed that the kidney index (Figure 2E), alanine aminotransferase (ALT, Figure 2F), aspartate aminotransferase (AST, Figure 2G), blood urea nitrogen (BUN, Figure 2H), and creatinine (CRE, Figure 2I) were all significantly elevated in the cisplatin group, indicating obvious hepatorenal toxicity. In contrast, none of these indices in the matrine groups differed significantly from the control group. H&E staining of the liver and kidney tissues in Supplementary Figure S2A further showed partial vacuolization of hepatocytes and edema of renal tubular epithelial cells and interstitium in the cisplatin group, whereas the liver, kidney, heart, and spleen tissues in the matrine groups were structurally normal. There were no significant differences in the liver and spleen coefficients among the groups (Supplementary Figure S2B,C). Thus, matrine inhibited osteosarcoma growth in vivo without obvious hepatorenal toxicity and was better tolerated than cisplatin.

### 3.3. Matrine Inhibits the Lung Metastasis of 143B Human Osteosarcoma Cells In Vivo

**Figure 3:**
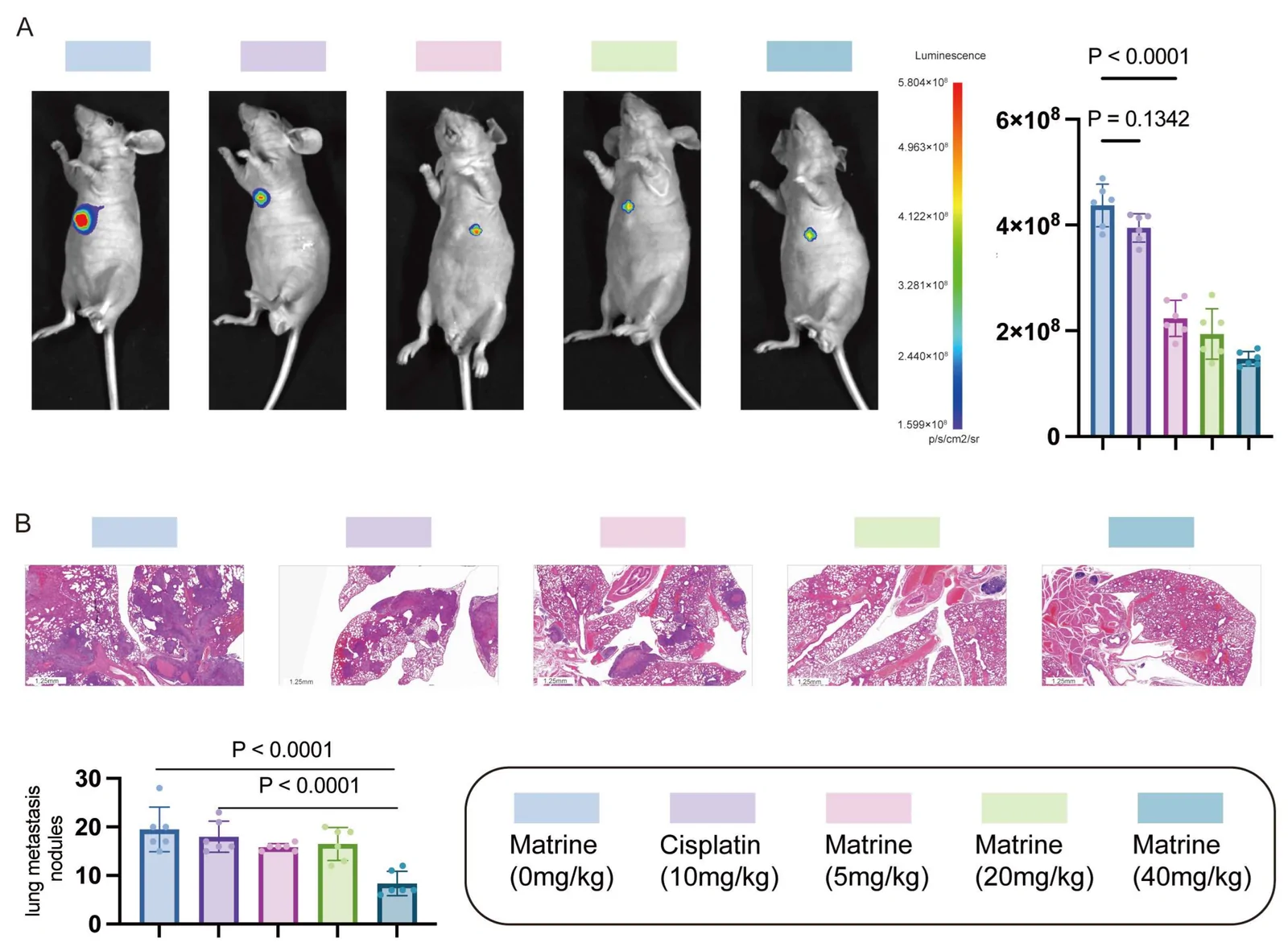
Matrine inhibits lung metastasis in a tail vein model. (**A**) In vivo bioluminescence imaging of the lungs and quantitative analysis of total flux. (**B**) H&E staining of lung tissues in the indicated groups. Scale bar, 1.25 mm. Data are presented as the mean ± SD. *n* = 6.

To further evaluate the inhibitory effect of matrine on distant metastasis of osteosarcoma, a lung metastasis model was established by injecting 143B cells through the tail vein. After 4 weeks of treatment, the fluorescence signal in the lungs was detected with an in vivo imaging system, and lung tissues were subjected to H&E staining and metastatic nodule counting. Bioluminescence imaging (Figure 3A) showed that the fluorescence signal was strongest in the lungs of control mice, indicating the highest pulmonary tumor burden. The fluorescence signals in the cisplatin group (10 mg/kg) and the matrine groups (5, 20, and 40 mg/kg) decreased in order, and the 40 mg/kg matrine group showed the lowest signal intensity. This indicated that matrine significantly inhibited the pulmonary colonization and proliferation of osteosarcoma cells. Quantitative analysis showed that the total flux of the control group was significantly higher than that of all treatment groups (*p* < 0.0001). The total flux of the 40 mg/kg matrine group was further lower than that of the cisplatin group (*p* = 0.1342). Although this difference did not reach statistical significance, it showed a trend toward a stronger effect than cisplatin. H&E staining of lung tissue (Figure 3B) showed extensive diffuse tumor cell infiltration and severe destruction of lung architecture in the control group. The extent of tumor infiltration decreased progressively in the cisplatin group and the matrine groups, and only scattered tumor cell clusters were observed in the 40 mg/kg matrine group. Metastatic nodule counts decreased in a dose-dependent manner across the matrine groups (all *p* < 0.0001), and the count in the 40 mg/kg group was lower than that in the cisplatin group. Taken together, matrine inhibited osteosarcoma lung metastasis in a dose-dependent manner, with efficacy comparable to that of cisplatin and a trend toward a stronger effect at 40 mg/kg.

### 3.4. Matrine Induces Mitochondrial Morphological Abnormalities and Dysfunction in 143B Human Osteosarcoma Cells In Vitro

**Figure 4:**
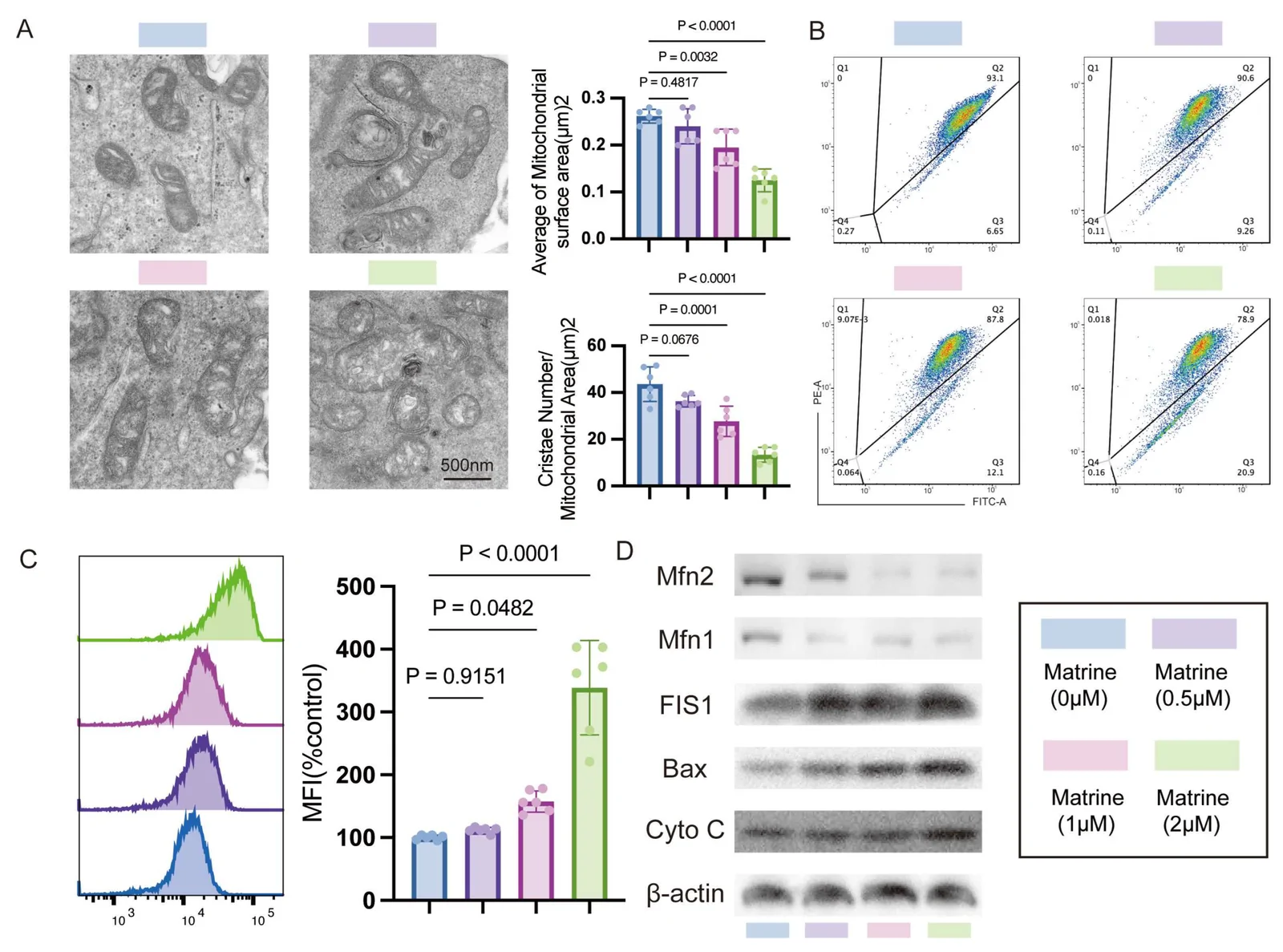
Matrine induces mitochondrial morphological abnormalities and dysfunction in 143B cells. (**A**) Transmission electron microscopy images of mitochondria in cells treated with the indicated concentrations of matrine. Scale bar, 500 nm. (**B**) Mitochondrial membrane potential detected by JC-1 staining and flow cytometry. (**C**) Intracellular ROS levels detected by DCFH-DA staining and flow cytometry. (**D**) Western blot analysis of Mfn1, Mfn2, FIS1, Bax, and cytochrome c expression, with β-actin as the loading control. Data are presented as the mean ± SD. *n* = 6.

To clarify the mitochondrial mechanisms underlying the inhibition of osteosarcoma cell proliferation by matrine, 143B human osteosarcoma cells were used. Mitochondrial ultrastructure was observed by transmission electron microscopy. The mitochondrial membrane potential was detected by JC-1 staining. Intracellular ROS levels were measured by flow cytometry. The expression of mitochondrial dynamics and apoptosis-related proteins was detected by Western blotting. Transmission electron microscopy (Figure 4A) showed that mitochondria in control cells were rod-shaped or oval, with dense, clear, and orderly cristae. After matrine treatment, the mitochondria gradually became short rods or spheres, and the cristae became broken or disordered or even disappeared. Some mitochondria showed vacuolization. The damage increased with rising matrine concentrations (0.5, 1, and 2 μM), indicating concentration-dependent disruption of mitochondrial morphology. Morphometric analysis further revealed that matrine treatment significantly reduced both the average mitochondrial surface area and the crista density (cristae number per mitochondrial area) in a concentration-dependent manner (*p* < 0.0001), quantitatively confirming mitochondrial fragmentation and crista degradation. Functional assays (Figure 4B,C) showed that the mitochondrial membrane potential decreased after matrine treatment, with further depolarization at higher concentrations. Flow cytometry showed that intracellular ROS levels increased in a concentration-dependent manner after matrine treatment (*p* < 0.0001), indicating increased overall intracellular oxidative stress. Western blotting (Figure 4D, Supplementary Figure S3) showed that matrine downregulated the mitochondrial fusion proteins Mfn2 and Mfn1 (*p* < 0.0001), upregulated the fission protein FIS1, and increased Bax and cytochrome c-associated apoptotic signaling (*p* < 0.0001). These findings indicated that matrine shifted mitochondrial dynamics toward fission, inhibiting fusion and promoting fission, and activated mitochondria-dependent apoptosis. Overall, matrine caused concentration-dependent mitochondrial damage in 143B cells, manifested by disrupted ultrastructure, membrane potential depolarization, elevated intracellular ROS levels, and mitochondrial dynamics imbalance.

### 3.5. Matrine Treatment Affects the PI3K/AKT Signaling Pathway

**Figure 5:**
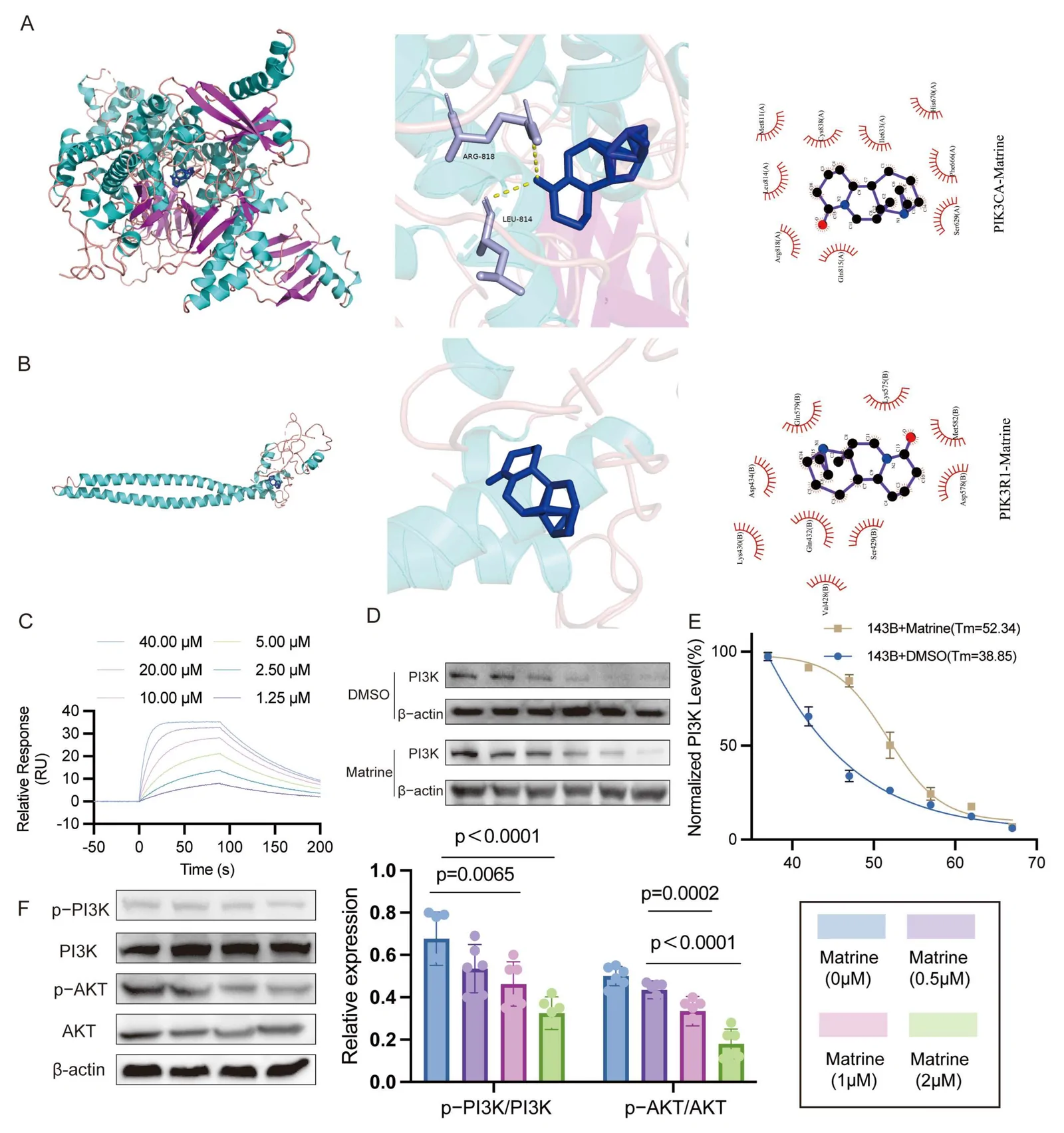
Matrine directly binds to PI3K and inhibits the PI3K/AKT signaling pathway. (**A**,**B**) Molecular docking of matrine with PI3K. (**C**) SPR analysis of matrine binding to PI3K. (**D**,**E**) CETSA showing the thermal stability of PI3K after matrine treatment. (**F**) Western blot analysis of p-PI3K, PI3K, p-AKT, and AKT after matrine treatment. *n* = 6.

To clarify the molecular targets and mechanisms of matrine against osteosarcoma, network pharmacology was used to predict the potential targets and key signaling pathways of matrine in treating osteosarcoma. Molecular docking, SPR, the CETSA, and Western blotting were used for verification. The network pharmacology results (Supplementary Figure S4A,B) showed the overlapping targets of matrine and osteosarcoma in a Venn diagram. The protein–protein interaction (PPI) network analysis identified key targets (Supplementary Figure S5). Gene Ontology (GO) and KEGG pathway enrichment analyses indicated that the PI3K/AKT signaling pathway was one of the core pathways through which matrine acts (Supplementary Figure S6A,B). Molecular docking (Figure 5A,B) showed that matrine could fit into the active pocket of PI3K and form a stable binding conformation, suggesting that matrine may directly target PI3K. SPR (Figure 5C) showed concentration-dependent binding of matrine to PI3K, with the response signal gradually increasing as the matrine concentration increased. This confirmed a direct interaction between the two. CETSA (Figure 5D,E) showed that matrine markedly increased the thermal stability of PI3K. The melting curve shifted markedly to the right compared with the dimethyl sulfoxide (DMSO) control, and the melting temperature (T_m_) value increased from 38.85 °C to 52.34 °C. This indicated that matrine could directly bind to and stabilize PI3K in cells. Western blotting (Figure 5F) showed that the p-PI3K/PI3K and p-AKT/AKT ratios were significantly decreased after matrine treatment compared with the control group (*p* < 0.0001), and the inhibition increased with rising concentrations (0.5 and 2 μM). Thus, matrine inhibited phosphorylation and activation of the PI3K/AKT signaling pathway. Collectively, network pharmacology, molecular docking, SPR, and CETSA confirmed that matrine directly binds to PI3K and blocks aberrant activation of the PI3K/AKT signaling pathway by inhibiting PI3K and AKT phosphorylation.

### 3.6. Matrine Inhibits Osteosarcoma Cell Proliferation and Invasion Through the PI3K/AKT Signaling Pathway

**Figure 6:**
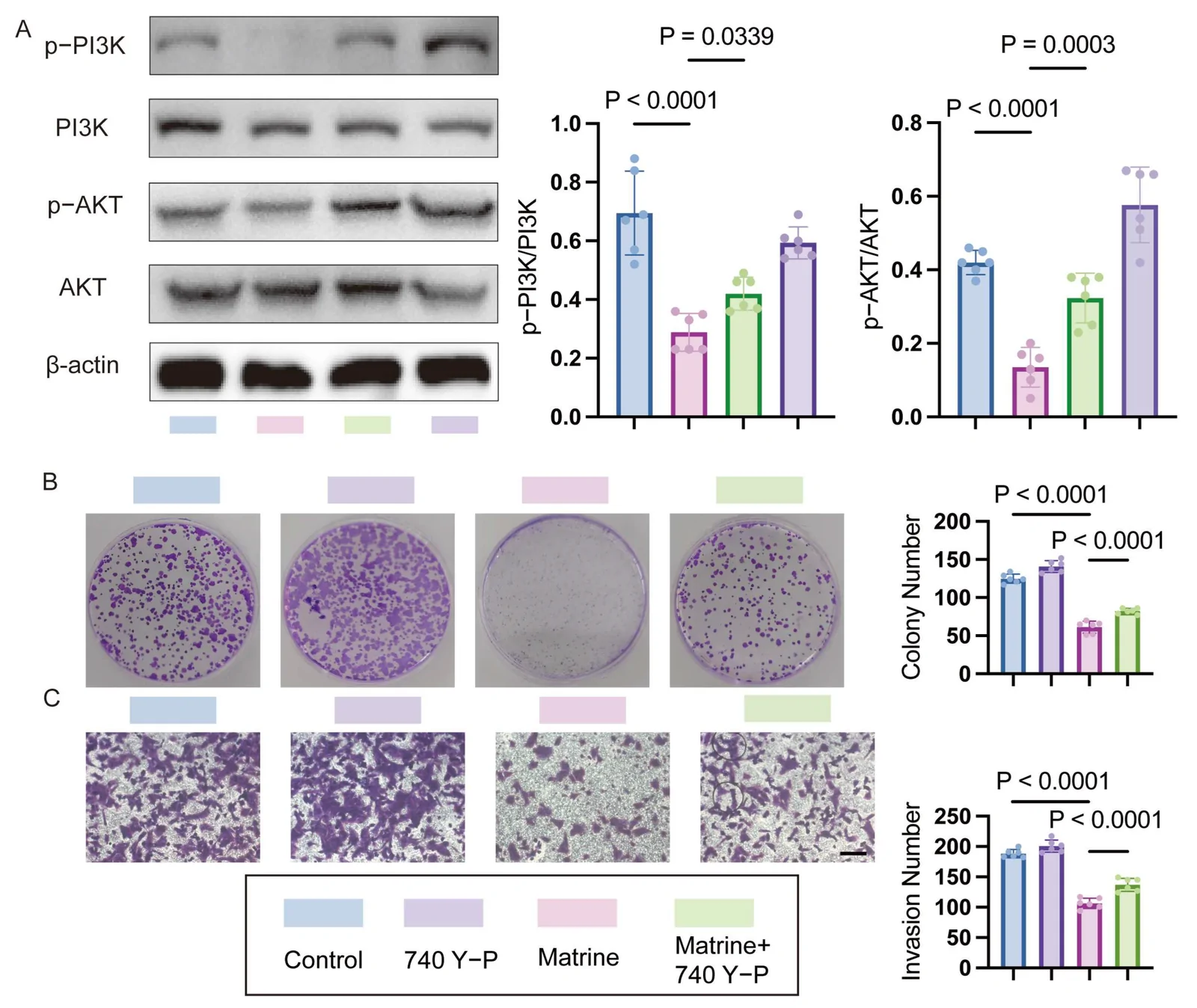
The PI3K agonist 740 Y-P partially reverses the effects of matrine on proliferation and invasion. (**A**) Colony formation assay. (**B**) Transwell invasion assay. (**C**) Western blot analysis of p-PI3K/PI3K and p-AKT/AKT. Cells were treated with matrine alone or in combination with 740 Y-P. Data are presented as the mean ± SD. *n* = 6.

To verify whether the inhibitory effects of matrine on the proliferation and invasion of osteosarcoma cells depend on the PI3K/AKT signaling pathway, a rescue experiment was performed with the PI3K agonist 740 Y-P in combination with matrine. In the colony formation assay (Figure 6A), the number of colonies was significantly reduced after matrine treatment compared with the control group (*p* < 0.0001). After coadministration of 740 Y-P, the colony number increased significantly compared with the matrine alone group (*p* < 0.0001). Thus, activating the PI3K/AKT pathway partially reversed the inhibition of proliferation by matrine. In the Transwell invasion assay (Figure 6B), the number of invading cells was significantly reduced after matrine treatment (*p* < 0.0001) and increased again after coadministration of 740 Y-P (*p* < 0.0001). Similarly, PI3K/AKT activation partially restored the invasion capacity of matrine-treated cells. Western blotting (Figure 6C) showed that the p-PI3K/PI3K and p-AKT/AKT ratios decreased after matrine treatment (*p* < 0.0001) and recovered after coadministration of 740 Y-P (*p* = 0.0003). This confirmed that 740 Y-P activated the PI3K/AKT pathway. Thus, the PI3K agonist 740 Y-P partially reversed the inhibitory effects of matrine on proliferation and invasion, indicating that these effects depend partly on the PI3K/AKT signaling pathway.

### 3.7. Matrine Inhibits Mitochondrial Function Through the PI3K/AKT Signaling Pathway

**Figure 7:**
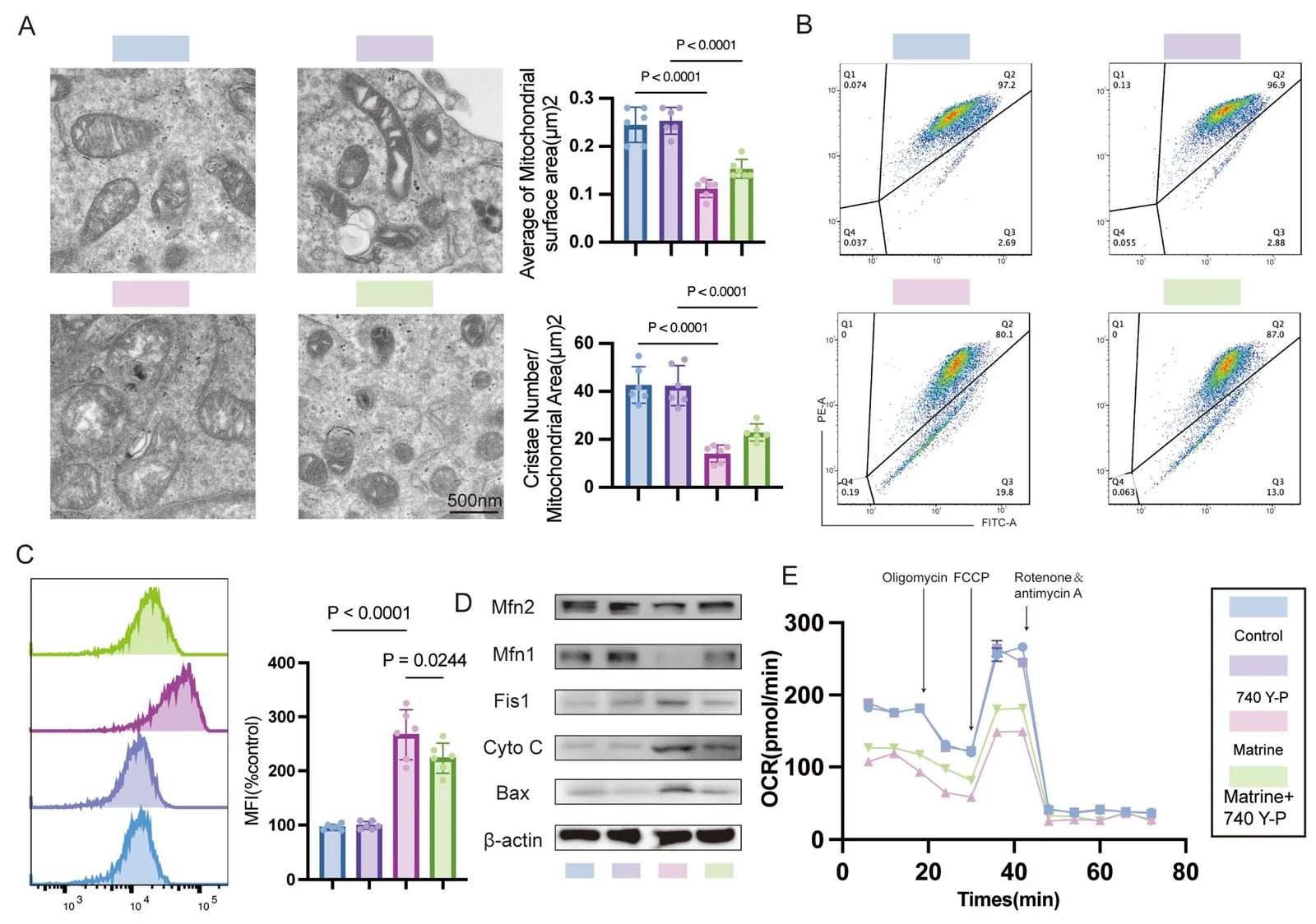
The PI3K agonist 740 Y-P partially reverses matrine-induced mitochondrial dysfunction. (**A**) Representative transmission electron microscopy (TEM) images of mitochondrial morphology in 143B cells treated with matrine alone or in combination with 740 Y-P. Scale bar = 500 nm. Quantification of mitochondrial surface area and crista density per mitochondrial area is shown in the bar graphs. (**B**) Flow cytometry analysis of mitochondrial membrane potential (ΔΨm). (**C**) Flow cytometry analysis of ROS levels. (**D**) Western blot analysis of Mfn1, Mfn2, FIS1, Bax, and cytochrome c expression. (**E**) Seahorse XF mitochondrial stress test. The OCR curve shows the response to sequential addition of oligomycin, FCCP, and rotenone/antimycin A. Bar graphs show basal OCR, maximal respiration, ATP production, and spare respiratory capacity. Data are presented as the mean ± SD. *n* = 6.

To verify whether the inhibitory effect of matrine on mitochondrial function depends on the PI3K/AKT signaling pathway, a rescue experiment was performed with the PI3K agonist 740 Y-P in combination with matrine. The mitochondrial membrane potential, ROS levels, and the expression of mitochondrial dynamics and apoptosis-related proteins were examined. Matrine-induced mitochondrial swelling and fragmentation were rescued by 740 Y-P (Figure 7A). Morphometric analysis showed that matrine significantly reduced both mitochondrial surface area and crista density (*p* < 0.0001), and these changes were partially reversed by 740 Y-P cotreatment, confirming the protective effect of PI3K/AKT activation on mitochondrial ultrastructure. Flow cytometry (Figure 7B,C) showed that the mitochondrial membrane potential decreased and ROS levels increased after matrine treatment (*p* < 0.0001). These changes were partially reversed after coadministration of 740 Y-P (*p* = 0.0244), indicating that activation of the PI3K/AKT signaling pathway could partially rescue the mitochondrial dysfunction induced by matrine. Western blotting (Figure 7D, Supplementary Figure S7) showed that matrine downregulated the mitochondrial fusion proteins Mfn1 and Mfn2 (*p* < 0.0001) and upregulated the mitochondrial fission protein FIS1 and Bax and cytochrome c-associated apoptotic signaling (*p* < 0.0001). After coadministration of 740 Y-P, the expression of Mfn1 and Mfn2 recovered, whereas that of FIS1, Bax, and cytochrome c decreased. This indicated that PI3K/AKT activation reversed the mitochondrial dynamics imbalance and apoptosis activation induced by matrine. To assess mitochondrial respiratory function, a Seahorse XF mitochondrial stress test was performed. Matrine treatment markedly reduced the basal OCR and maximal respiratory capacity, whereas cotreatment with 740 Y-P partially restored both parameters (Figure 7E), indicating that inhibition of the PI3K/AKT pathway impairs mitochondrial respiration and that its activation can rescue this defect. Matrine induced mitochondrial dysfunction in osteosarcoma cells by inhibiting the PI3K/AKT signaling pathway, manifested by membrane potential depolarization, ROS accumulation, mitochondrial dynamics imbalance, and mitochondria-dependent apoptosis.

### 3.8. Rescue of the PI3K/AKT Pathway Attenuates the Inhibition of Mitochondrial Function and Lung Metastasis by Matrine

**Figure 8:**
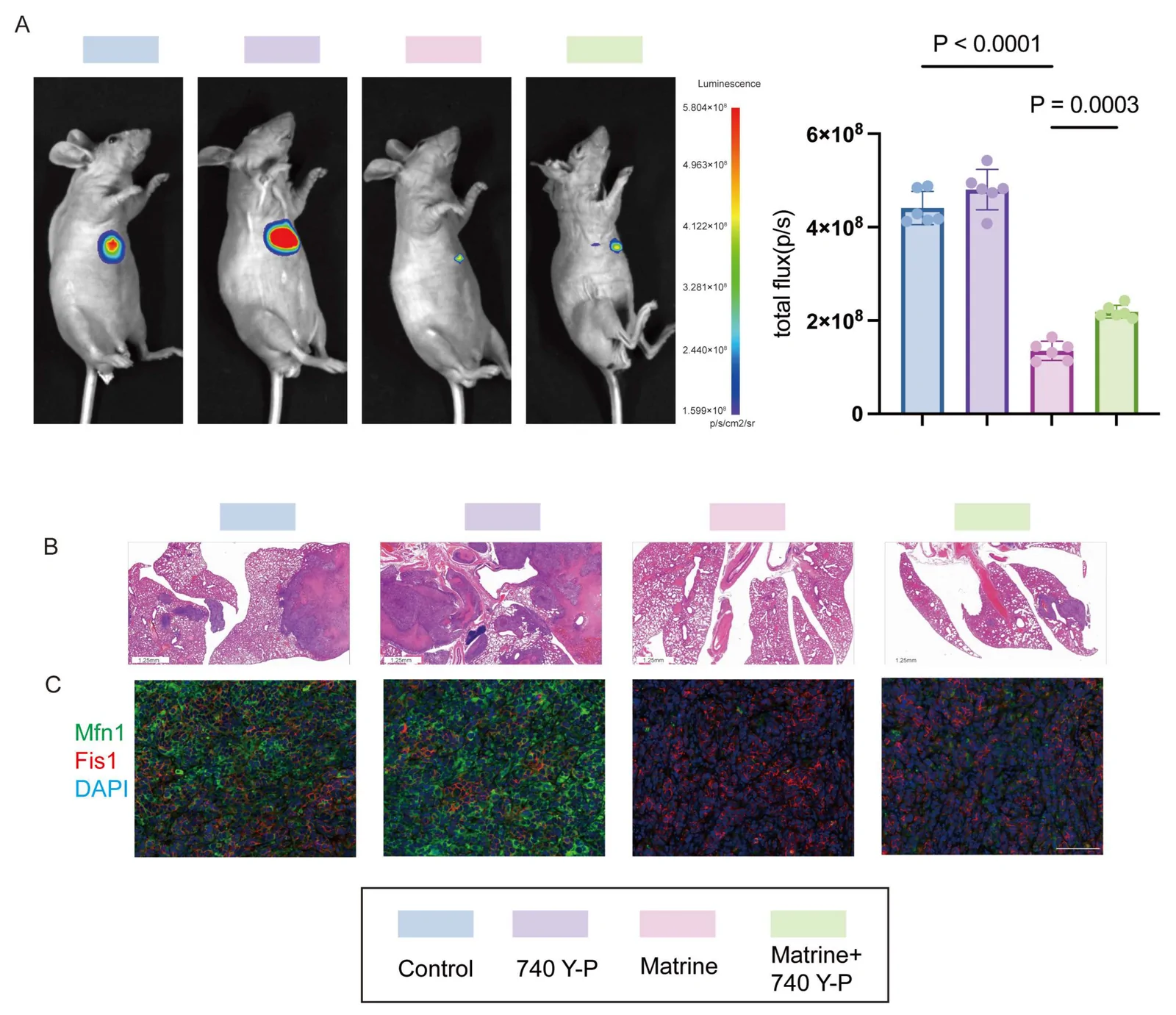
The PI3K agonist 740 Y-P attenuates the antimetastatic effect of matrine in vivo. (**A**) In vivo bioluminescence imaging of the lungs. (**B**) H&E staining of lung tissues. Scale bar, 1.25 mm. (**C**) Immunofluorescence staining of Mfn1 and FIS1 in lung metastatic foci. Scale bar, 100 μm. Mice were treated with matrine alone or in combination with 740 Y-P. Data are presented as the mean ± SD. *n* = 6.

To further verify that the in vivo effect of matrine against lung metastasis depends on the PI3K/AKT signaling pathway, a rescue experiment was performed with the PI3K agonist 740 Y-P in combination with matrine using the 143B-Luc lung metastasis model. In vivo imaging (Figure 8A) showed that the thoracic bioluminescence signal was significantly lower after matrine treatment than in the control group, confirming that matrine effectively inhibited the pulmonary colonization of osteosarcoma cells in vivo. After coadministration of 740 Y-P, the bioluminescence signal that had been suppressed by matrine partially recovered (*p* = 0.0003). This indicated that exogenous activation of the PI3K/AKT signaling pathway partially weakened the ability of matrine to inhibit lung metastasis in vivo. H&E staining of lung tissue (Figure 8B) showed that the lung metastatic foci in the matrine alone group were markedly reduced and the alveolar structure was relatively intact, whereas the metastatic foci area in the matrine plus 740 Y-P group was larger than that in the matrine group. This indicated partial restoration of pulmonary metastatic colonization. Immunofluorescence (Figure 8C) showed that, compared with the control group, the fluorescence of the mitochondrial fusion protein Mfn1 in the lung metastatic foci of the matrine group was markedly weaker, whereas that of the mitochondrial fission protein FIS1 was markedly stronger (*p* < 0.0001). This reflected a profission and antifusion change. After coadministration of 740 Y-P, the Mfn1 fluorescence recovered and the FIS1 fluorescence weakened, indicating that PI3K/AKT activation partially reversed the regulation of mitochondrial dynamics by matrine in vivo. Therefore, the in vivo effects of matrine on osteosarcoma lung metastasis and mitochondrial dynamics depend partly on inhibition of the PI3K/AKT signaling pathway; when this pathway is exogenously activated, the antimetastatic effect of matrine is correspondingly weakened.

## 4. Discussion

Osteosarcoma is the most common primary malignant bone tumor in adolescents, and its clinical prognosis is closely related to the occurrence of lung metastasis [1,23]. Neoadjuvant chemotherapy combined with surgical resection has improved the five-year survival rate of patients with localized disease to 65% to 75%. However, once lung metastasis occurs, the five-year survival rate drops sharply to about 20%, with no significant improvement over the past three decades [1]. The survival benefit of resection of lung metastatic lesions remains controversial [5,6,7]. In this study, we systematically evaluated the antitumor activity of matrine against human osteosarcoma cells in vitro and in vivo. In vitro, matrine inhibited the proliferation, colony formation, and invasion of 143B, U2OS, and MG63 cells in a concentration-dependent manner, with 143B cells being the most sensitive. In vivo, matrine significantly suppressed tumor growth in the subcutaneous xenograft model and reduced the lung metastatic burden in the tail vein lung metastasis model. The in vivo imaging and histopathological results were consistent, and no obvious organ toxicity was observed at the tested doses. These data demonstrate that matrine has activity against osteosarcoma and its lung metastasis at both the cellular and animal levels, supporting its antimetastatic application.

Mitochondrial dynamics refers to the precise balance between mitochondrial fusion and fission. This balance is essential for maintaining mitochondrial function, cell metabolism, and survival, and its disruption has been recognized as an important feature of tumor biology [20,24]. To reveal the subcellular mechanisms by which matrine inhibits the proliferation and metastasis of osteosarcoma cells, we focused on mitochondria. Transmission electron microscopy showed that the mitochondria of osteosarcoma cells were markedly swollen with broken cristae and vacuolization after matrine treatment, representing typical structural damage. JC-1 flow cytometry and ROS assays confirmed the reduction in the mitochondrial membrane potential and the accumulation of ROS. Protein analysis further showed that matrine upregulated the mitochondrial fission protein FIS1 and Bax and cytochrome c-associated apoptotic signaling while downregulating the mitochondrial fusion proteins Mfn1 and Mfn2. This suggested that matrine shifted the mitochondrial dynamics balance toward fission, which increased mitochondrial outer membrane permeability, promoted cytochrome c release, and activated the mitochondria-dependent apoptosis pathway. These findings support an association between matrine-induced mitochondrial dysfunction, altered mitochondrial dynamics, and activation of apoptotic signaling.

Lung metastasis is the main cause of treatment failure and death in patients with osteosarcoma. The metastatic cascade involves tumor cell detachment from the primary site, intravasation, survival in the circulation, extravasation, and colonization at distant sites [25]. We found that matrine inhibited the invasion of 143B cells at concentrations as low as 1 μM and blocked the formation of lung metastatic nodules in vivo in a dose-dependent manner. Several lines of evidence link mitochondrial fission to cell migration. Fission proteins can redistribute mitochondria to the leading edge of lamellipodia and focal adhesions, providing local energy for actin polymerization and cell movement [26,27]. Previous studies have also reported enhanced mitochondrial fission and mitochondrial redistribution to the leading edge in metastatic tumor cells, supporting the local energy supply required for cell migration [26,28]. Therefore, the mitochondrial fusion arrest and excessive fission induced by matrine (upregulation of FIS1 and downregulation of Mfn1/Mfn2) may directly weaken the motility and invasion potential of osteosarcoma cells. In addition, matrine has been shown to suppress EMT in breast cancer through the LINC01116/miR-9-5p/ITGB1 axis [13]. This suggests that its antimetastatic effect may involve several mechanisms, including mitochondrial regulation, cytoskeletal remodeling, and EMT inhibition. Compared with conventional chemotherapy that is often accompanied by severe systemic toxicity, matrine, as a natural alkaloid, has the advantages of multiple targets and low toxicity, and its in vivo safety has been preliminarily verified in multiple studies.

The PI3K/AKT signaling pathway is frequently aberrantly activated in osteosarcoma and is closely related to tumor proliferation, apoptosis resistance, metastasis, and chemoresistance [19,29,30]. Through network pharmacology prediction, molecular docking, SPR, and the CETSA, we confirmed that matrine directly binds to PI3K and inhibits the phosphorylation of PI3K and AKT in a concentration-dependent manner. The rescue experiments further showed that the PI3K agonist 740 Y-P only partially reversed the inhibitory effects of matrine on proliferation, invasion, and mitochondrial damage. This incomplete rescue suggested that the effect of matrine against osteosarcoma does not entirely depend on the PI3K/AKT pathway. Previous studies have shown that matrine can also inhibit osteosarcoma proliferation through the MAPK/ERK pathway [18] and suppress malignant phenotypes through the PI3K/AKT axis in gallbladder cancer [31]. We therefore speculate that matrine may coordinate the regulation of mitochondrial dynamics and the metastatic phenotype by simultaneously modulating multiple pathways, including PI3K/AKT and MAPK/ERK, which is consistent with the ability of natural products to act on multiple targets. Notably, matrine upregulated the fission protein FIS1 and downregulated the fusion proteins Mfn1/Mfn2 after inhibiting PI3K/AKT. This regulatory direction deserves further discussion. Previous studies have shown that PI3K/AKT activation can promote mitochondrial fission through the mechanistic target of rapamycin (mTOR) pathway [20]. However, other evidence indicates that the regulation of mitochondrial dynamics proteins by PI3K/AKT is cell type-specific and context-dependent, and its effects at the transcriptional and post-translational levels may differ. Whether PI3K/AKT inhibits FIS1 transcription through a negative feedback mechanism in osteosarcoma, or whether acute PI3K/AKT inhibition indirectly upregulates fission proteins through compensatory stress pathways, remains to be clarified.

Several limitations should be acknowledged. The mechanistic validation relied mainly on a single cell line, 143B, and, although this line was the most sensitive to matrine and performed reliably in vivo, the findings should be confirmed in additional osteosarcoma cell lines and patient-derived xenograft models. Pathway rescue was performed with the pharmacological agonist 740 Y-P rather than genetic approaches such as PI3K small interfering RNA (siRNA) or clustered regularly interspaced short palindromic repeats (CRISPR) silencing; future studies should therefore use gene silencing to establish the causal role of PI3K/AKT more rigorously. The tail vein lung metastasis model reproduces hematogenous dissemination and pulmonary colonization but omits the earlier steps of invasion and intravasation, so orthotopic or spontaneous metastasis models are needed for complementary validation. Whether matrine also acts through the tumor immune microenvironment, for example by modulating macrophage polarization or natural killer cell activity, remains to be explored. Finally, the low oral bioavailability and short half-life of matrine remain obstacles to clinical translation, and nanodelivery systems or matrine derivatives may help overcome them.

## 5. Conclusions

Matrine suppresses osteosarcoma lung metastasis by targeting the PI3K/AKT pathway to induce mitochondrial dysfunction and may be a useful therapeutic agent.

## Data Availability

Raw data are available from the corresponding author upon reasonable request.

## Supplementary Materials

The following supporting information can be downloaded at: https://www.mdpi.com/article/10.3390/cancers18203381/s1. Figure S1. Selective cytotoxicity of matrine toward osteosarcoma cells over non-malignant bone-derived cells; Figure S2. H&E staining of major organs. Representative H&E staining of the liver, kidney, heart, and spleen in the indicated groups; Figure S3. Quantification of Western blot analysis of mitochondrial dynamics and apoptosis related proteins. Statistical analysis of the relative expression of Mfn1, Mfn2, FIS1, Bax, and cytochrome c in 143B cells treated with matrine. Data are presented as the mean ± SD.; Figure S4. Network pharmacology analysis of matrine against osteosarcoma; Figure S5. PPI network of the core targets; Figure S6. Gene Ontology (GO) and KEGG pathway enrichment analyses; Figure S7. Quantification of Western blot analysis in the rescue experiment. Statistical analysis of the relative expression of Mfn1, Mfn2, FIS1, Bax, and cytochrome c in the rescue experiment with 740 Y-P. Data are presented as the mean ± SD; Table S1. Antibody Information Table; File S1. Raw western blot image.

## Abbreviations

**Abbreviation**	**Full name**
3R	replacement, reduction, refinement
AKT	protein kinase B
ALT	alanine aminotransferase
ANOVA	analysis of variance
AST	aspartate aminotransferase
Bax	Bcl-2-associated X protein
BCA	bicinchoninic acid
BP	biological process
BUN	blood urea nitrogen
CC	cellular component
CCK-8	Cell Counting Kit-8
CETSA	cellular thermal shift assay
CRE	creatinine
CRISPR	clustered regularly interspaced short palindromic repeats
DAVID	Database for Annotation, Visualization and Integrated Discovery
DCFH-DA	2′,7′-dichlorodihydrofluorescein diacetate
DMEM	Dulbecco’s modified Eagle’s medium
DMSO	dimethyl sulfoxide
ECL	enhanced chemiluminescence
EMT	epithelial–mesenchymal transition
ERK	extracellular signal-regulated kinase
FBS	fetal bovine serum
FIS1	mitochondrial fission 1 protein
GO	Gene Ontology
H&E	hematoxylin and eosin
IC50	half-maximal inhibitory concentration
JC-1	5,5′,6,6′-tetrachloro-1,1′,3,3′-tetraethylbenzimidazolylcarbocyanine iodide
KEGG	Kyoto Encyclopedia of Genes and Genomes
MAPK	mitogen-activated protein kinase
MF	molecular function
MFI	mean fluorescence intensity
Mfn1	mitofusin 1
Mfn2	mitofusin 2
mTOR	mechanistic target of rapamycin
OMIM	Online Mendelian Inheritance in Man
PBS	phosphate-buffered saline
PI3K	phosphatidylinositol 3-kinase
PPI	protein–protein interaction
PVDF	polyvinylidene fluoride
RIPA	radioimmunoprecipitation assay
ROS	reactive oxygen species
SD	standard deviation
SDS-PAGE	sodium dodecyl sulfate–polyacrylamide gel electrophoresis
siRNA	small interfering RNA
SPF	specific pathogen-free
SPR	surface plasmon resonance
STRING	Search Tool for the Retrieval of Interacting Genes/Proteins
TBST	Tris-buffered saline with Tween 20
TCMSP	Traditional Chinese Medicine Systems Pharmacology Database and Analysis Platform
Tm	melting temperature
TTD	Therapeutic Target Database
UniProt	Universal Protein Resource

## References

[1] Mirabello L., Troisi R.J., Savage S.A. (2009). Osteosarcoma incidence and survival rates from 1973 to 2004: Data from the Surveillance, Epidemiology, and End Results Program. Cancer.

[2] Beird H.C., Bielack S.S., Flanagan A.M., Gill J., Heymann D., Janeway K.A., Livingston J.A., Roberts R.D., Strauss S.J., Gorlick R. (2022). Osteosarcoma. Nat. Rev. Dis. Primers.

[3] Zhang S., He L., Shang J., Chen L., Xu Y., Chen X., Li X., Jiao Q., Jin S., Hu X. (2022). Carvacrol Suppresses Human Osteosarcoma Cells via the Wnt/β-Catenin Signaling Pathway. Anticancer Agents Med. Chem..

[4] Gu Z., Zhang S., Li G., Hu X., Xu N., Wang Y., Ruan J., Ma W., Chen H. (2025). Relationship between Ki-67 and P53 immunohistochemical expression and osteosarcoma chemotherapy sensitivity and long-term prognosis. Am. J. Transl. Res..

[5] Boam T., Rogoyski B.G., Jawaid W., Losty P.D. (2024). Do Children With Osteosarcoma Benefit From Pulmonary Metastasectomy?: A Systematic Review of Published Studies and “Real World” Outcomes. Ann. Surg..

[6] Ning B., Luo X., Wei G., Feng W., Zou Z., Li S., Xiong L., Miao J., Tang H., Li F. (2026). Pulmonary metastasectomy and survival in osteosarcoma: A systematic review and meta-analysis of surgery-related prognostic factors. World J. Surg. Oncol..

[7] Nakabachi K., Shimizu H., Matsuoka M., Soma T., Adachi H., Mizukami Y., Iwasaki N., Iwata A., Hiraga H. (2026). Clinical Outcomes and Surgical Procedures for Patients With Osteosarcoma and Metachronous Lung Metastasis: A Chronological Analysis. J. Surg. Oncol..

[8] Liang J., Qiao G., Zhang Y., Yuan Y., Liu Z., Jiang Y., Zhang Y., Deng Z., Yu L., Lin H. (2025). Ailanthone targets the KMT2A-MEN1 complex to suppress lung metastasis of osteosarcoma. Phytomedicine.

[9] Xu W., Wang Z., Liu T., Ma X., Jiao M., Zhao W., Yu L., Hua Y., Cai Z., Li J. (2024). Eurycomanone inhibits osteosarcoma growth and metastasis by suppressing GRP78 expression. J. Ethnopharmacol..

[10] Chen F., Pan Y., Xu J., Liu B., Song H. (2022). Research progress of matrine’s anticancer activity and its molecular mechanism. J. Ethnopharmacol..

[11] Xiong Z., Pu J. (2025). Matrine and Its Derivatives: Multi-Pathway Regulation in Cancer Therapy. Cancer Manag. Res..

[12] Jin J., Fan Z., Long Y., Li Y., He Q., Yang Y., Zhong W., Lin D., Lian D., Wang X. (2024). Matrine induces ferroptosis in cervical cancer through activation of piezo1 channel. Phytomedicine.

[13] Ren L., Fang Z., Xu J., Wu X., Zhang Y., Cai H., Han Z. (2025). Matrine Inhibits Breast Cancer Cell Proliferation and Epithelial-Mesenchymal Transition Through Regulating the LINC01116/miR-9-5p/ITGB1 Axis. Balk. Med. J..

[14] Huang Z., Li H., Li Q., Chen X., Liu R., Chang X. (2023). Matrine suppresses liver cancer progression and the Warburg effect by regulating the circROBO1/miR-130a-5p/ROBO1 axis. J. Biochem. Mol. Toxicol..

[15] Dong Y.F., Shang T. (2025). Matrine Alleviates Oxidative Stress and Inflammation in Colon Cancer by Activating the Nrf2 Pathway. Turk. J. Gastroenterol..

[16] Meng D., Cui Z., Li L., Shang Q., Chen X. (2025). Matrine in cancer therapy: Antitumor mechanisms and nano-delivery strategies. Front. Pharmacol..

[17] Almukram A.M.A., Al-hussaniy H.A., Jabarah M.A., Al-Abdeen S.H.Z. (2025). MDM2 antagonists and p53-targeting therapies in cancer: Clinical applications, adverse effects, and resistance mechanisms. Med. Pharm. J..

[18] Huang X., Zeng J., Ruan S., Lei Z., Zhang J., Cao H. (2024). The use of matrine to inhibit osteosarcoma cell proliferation via the regulation of the MAPK/ERK signaling pathway. Front. Oncol..

[19] Zhang J., Yu X.H., Yan Y.G., Wang C., Wang W.J. (2015). PI3K/Akt signaling in osteosarcoma. Clin. Chim. Acta.

[20] Senft D., Ronai Z.e.A. (2016). Regulators of mitochondrial dynamics in cancer. Curr. Opin. Cell Biol..

[21] Huang Z., Liu C.A., Cai P.Z., Xu F.P., Zhu W.J., Wang W.W., Jiang H.P. (2020). Omega-3PUFA Attenuates MNU-Induced Colorectal Cancer in Rats by Blocking PI3K/AKT/Bcl-2 Signaling. Onco Targets Ther..

[22] Li D., Guo Y.Y., Cen X.F., Qiu H.L., Chen S., Zeng X.F., Zeng Q., Xu M., Tang Q.Z. (2022). Lupeol protects against cardiac hypertrophy via TLR4-PI3K-Akt-NF-κB pathways. Acta Pharmacol. Sin..

[23] Bashir A., Ismail A., Mavadia A., Ghose A., Ovsepian S.V., Boussios S. (2025). Pathobiology and Molecular Pathways Implicated in Osteosarcoma Lung Metastasis: A Scoping Review. Technol. Cancer Res. Treat..

[24] Wu Z., Xiao C., Long J., Huang W., You F., Li X. (2024). Mitochondrial dynamics and colorectal cancer biology: Mechanisms and potential targets. Cell Commun. Signal.

[25] Yu J., Lu W., Chen Y., Li Y., Yang J., Cai Z., Li L., Fang K., Guo Y., Kang H. (2025). Butyrate derived from intratumoral microbiome facilitates pulmonary metastasis in osteosarcoma. Pharmacol. Res..

[26] Zhao J., Zhang J., Yu M., Xie Y., Huang Y., Wolff D.W., Abel P.W., Tu Y. (2013). Mitochondrial dynamics regulates migration and invasion of breast cancer cells. Oncogene.

[27] Minarrieta L., Annis M.G., Audet-Delage Y., Kuasne H., Pacis A., St-Louis C., Nowakowski A., Biondini M., Khacho M., Park M. (2024). Mitochondrial elongation impairs breast cancer metastasis. Sci. Adv..

[28] Poole L.P., Macleod K.F. (2021). Mitophagy in tumorigenesis and metastasis. Cell. Mol. Life Sci..

[29] Sadrkhanloo M., Paskeh M.D.A., Hashemi M., Raesi R., Bahonar A., Nakhaee Z., Entezari M., Beig Goharrizi M.A.S., Salimimoghadam S., Ren J. (2023). New emerging targets in osteosarcoma therapy: PTEN and PI3K/Akt crosstalk in carcinogenesis. Pathol. Res. Pract..

[30] Zhou Z., Li J., Wang Q., Zhang S., Li Y., Jin S., Wang Z., Chen J., Yang W., Liu T. (2025). CUDC-907 inhibits osteosarcoma through upregulation of PTX3 and inhibition of PI3K-AKT signaling. Eur. J. Pharmacol..

[31] Mo R.L., Li Z., Zhang P., Sheng M.H., Han G.C., Sun D.Q. (2024). Matrine inhibits invasion and migration of gallbladder cancer via regulating the PI3K/AKT signaling pathway. Naunyn Schmiedebergs Arch. Pharmacol..

