# Supplementary material for: Matrine Suppresses Osteosarcoma Lung Metastasis by Targeting the PI3K/AKT Signaling Pathway to Induce Mitochondrial Dysfunction
Source: Cancers (Basel). 2026 Oct 19;18(20):3381. doi: 10.3390/cancers18203381 (PMC13702236; doi:10.3390/cancers18203381)
Supplement: Supplementary file 1 [file cancers-18-03381-s001.zip › cancers-4608535-supplementary.pdf]

Supplementary figures

Figure S1

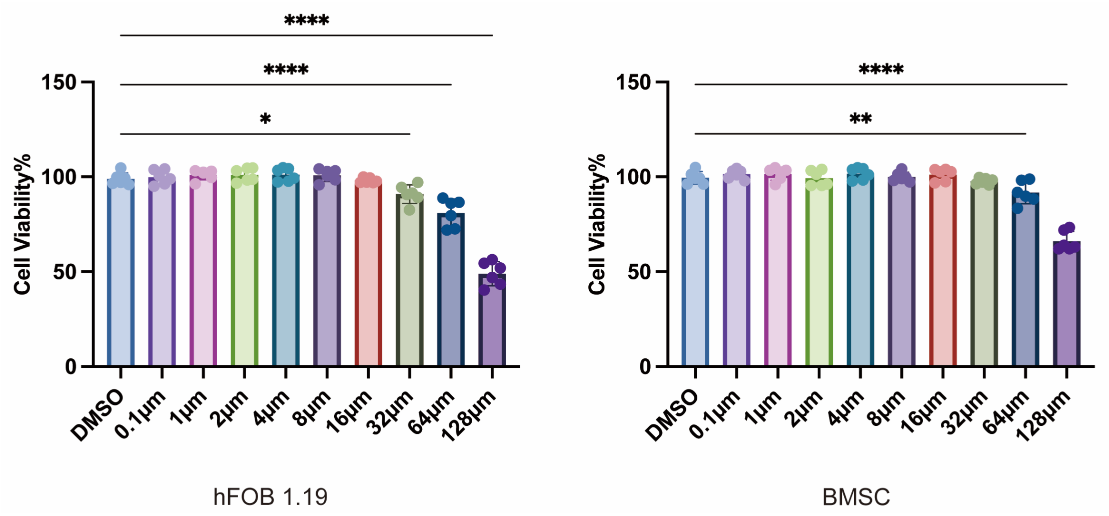

Figure S2

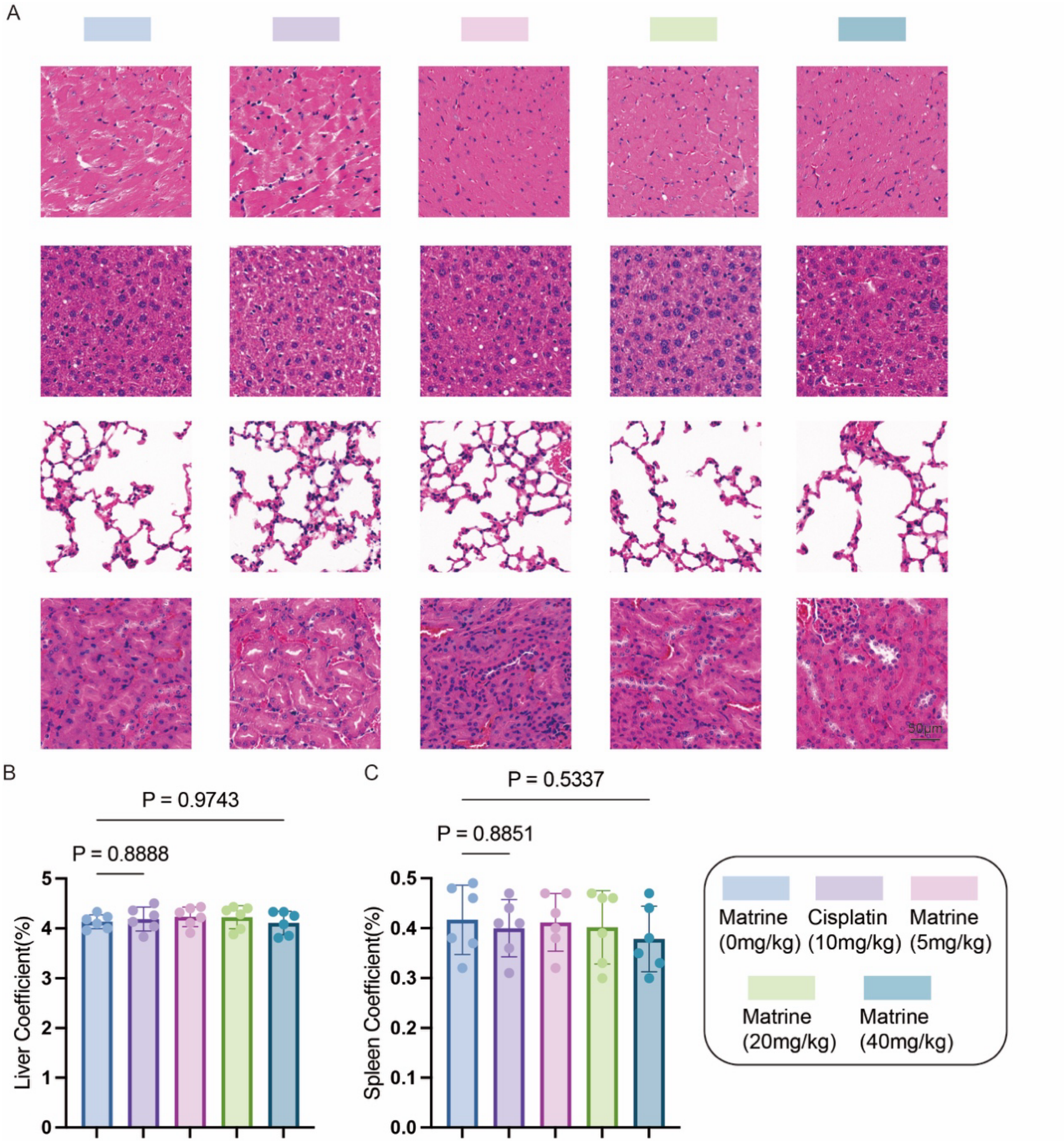

A
50μm
B
P = 0.9743
P = 0.8888
Liver Coefficient(%)
5
4
3
2
1
0
C
P = 0.5337
P = 0.8851
Spleen Coefficient(%)
0.5
0.4
0.3
0.2
0.1
0.0
Matrine (0mg/kg)
Cisplatin (10mg/kg)
Matrine (5mg/kg)
Matrine (20mg/kg)
Matrine (40mg/kg)

Figure S3

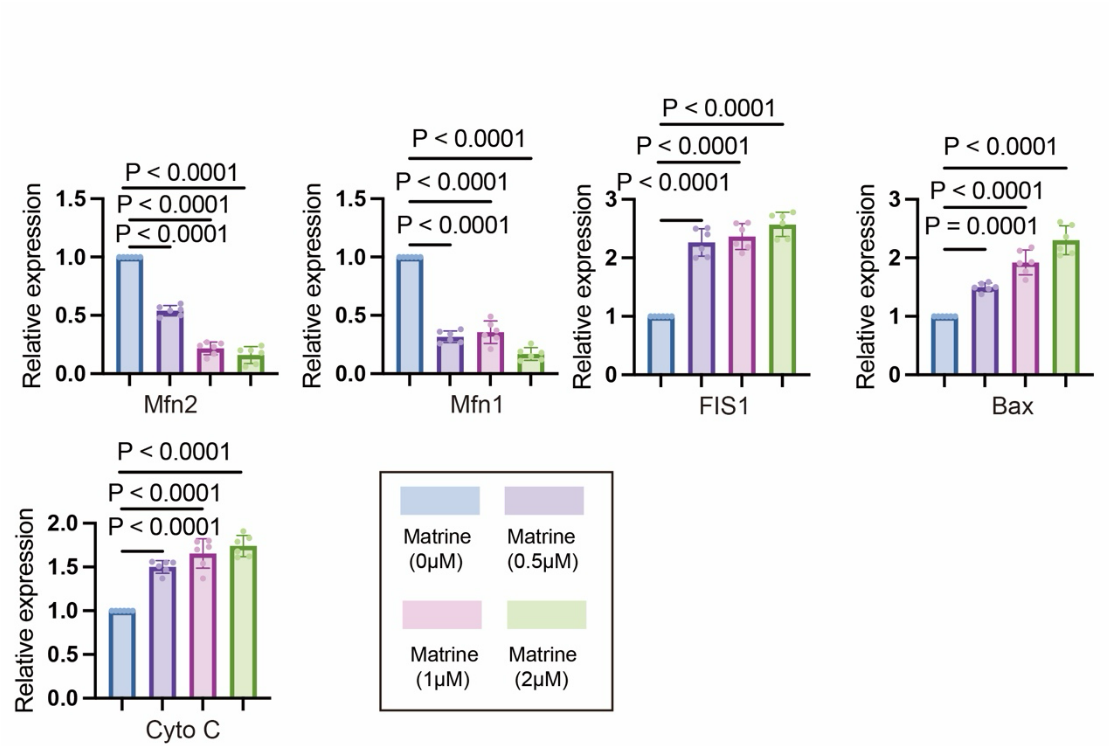
P < 0.0001
P < 0.0001
P < 0.0001
Relative expression
Mfn2
Mfn1
FIS1
Bax
P = 0.0001
Cyto C
Matrine (0μM)
Matrine (0.5μM)
Matrine (1μM)
Matrine (2μM)

Figure S4

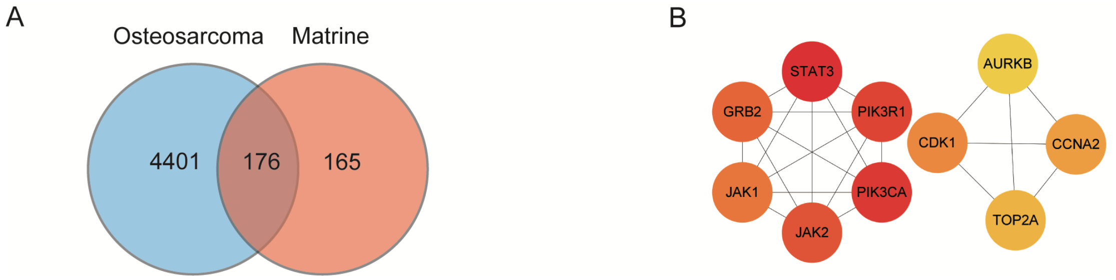
A
Osteosarcoma
Matrine
4401
176
165
B
STAT3
GRB2
PIK3R1
JAK1
PIK3CA
JAK2
AURKB
CDK1
CCNA2
TOP2A

Figure S5

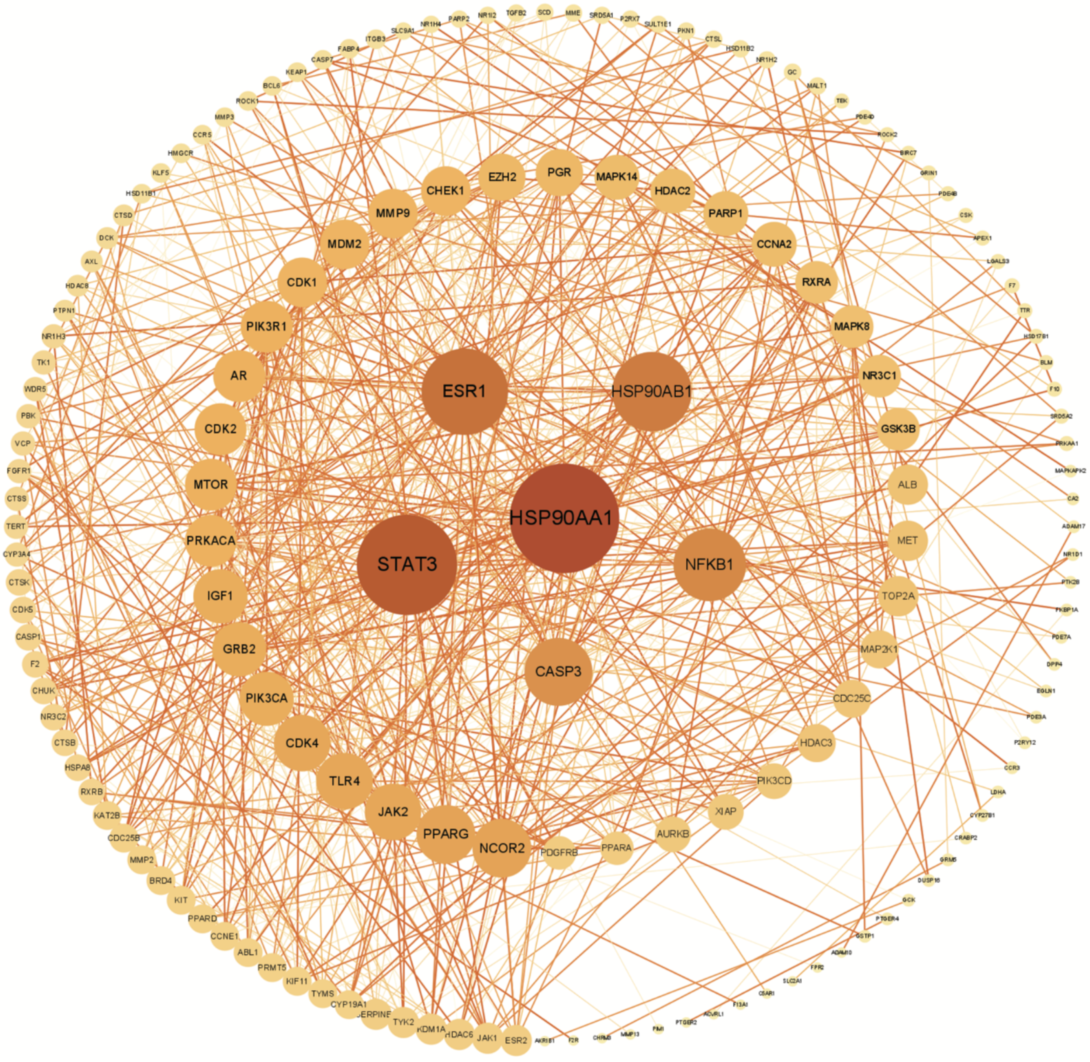

Figure S6

A

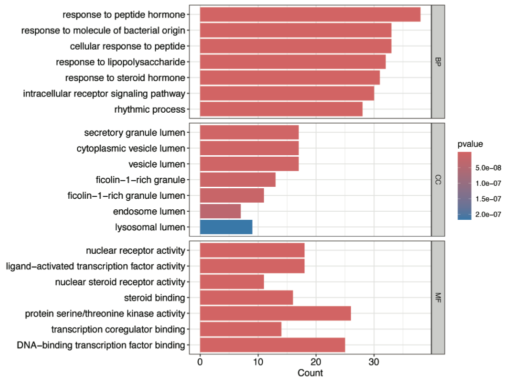

B

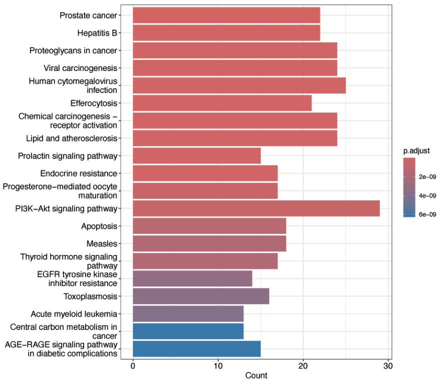

Figure S7

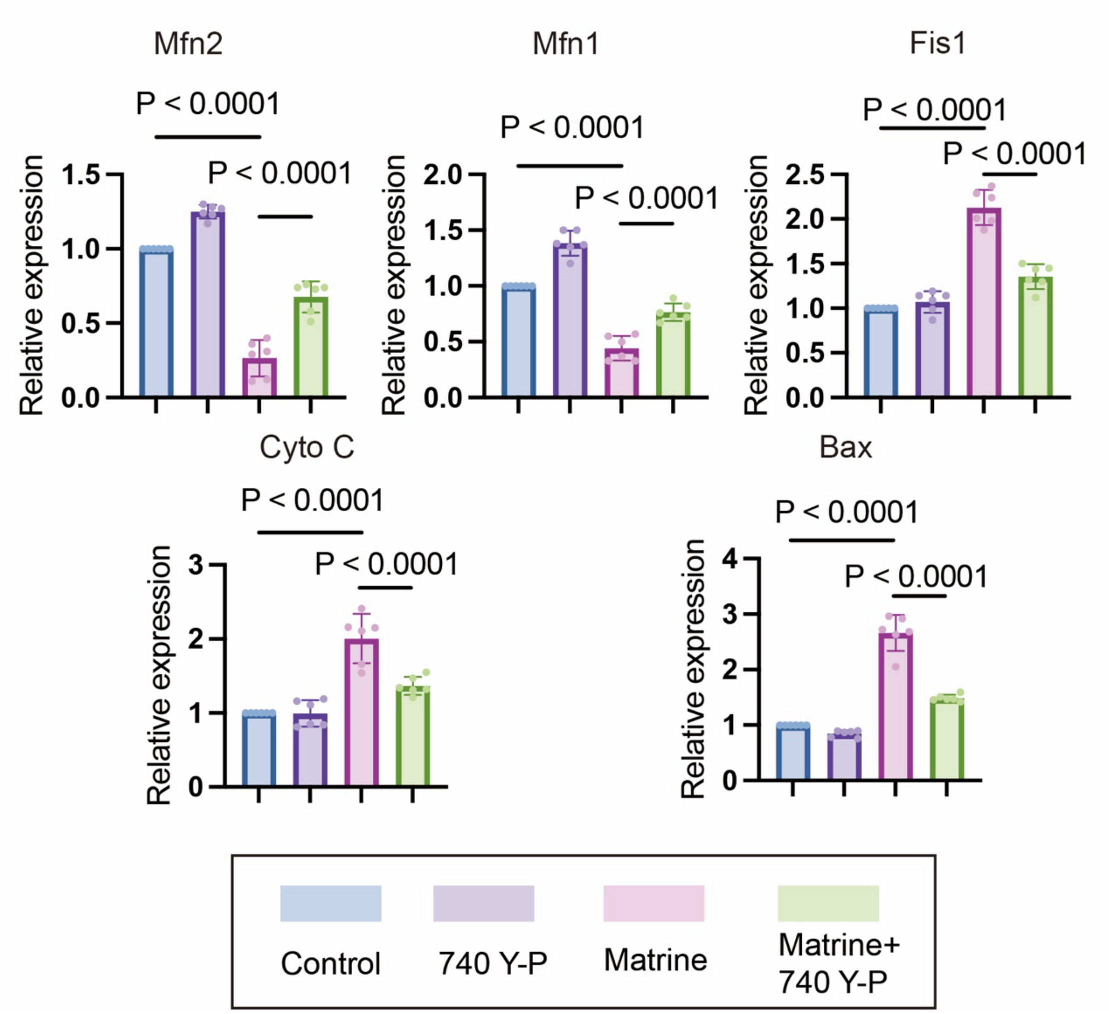

Mfn2
P < 0.0001
P < 0.0001
Relative expression
1.5
1.0
0.5
0.0
Mfn1
P < 0.0001
P < 0.0001
Relative expression
2.0
1.5
1.0
0.5
0.0
Fis1
P < 0.0001
P < 0.0001
Relative expression
2.5
2.0
1.5
1.0
0.5
0.0
Cyto C
P < 0.0001
P < 0.0001
Relative expression
3
2
1
0
Bax
P < 0.0001
P < 0.0001
Relative expression
4
3
2
1
0
Control
740 Y-P
Matrine
Matrine+ 740 Y-P

Supplementary Figure Legends

Figure S1 Selective cytotoxicity of matrine toward osteosarcoma cells over non-malignant bone-derived cells

Figure S2.H&E staining of major organs. Representative H&E staining of the liver, kidney, heart, and spleen in the indicated groups.

Figure S3. Quantification of Western blot analysis of mitochondrial dynamics and apoptosis related proteins. Statistical analysis of the relative expression of Mfn1, Mfn2, FIS1, Bax, and cytochrome c in 143B cells treated with matrine. Data are presented as the mean ± SD.

Figure S4. Network pharmacology analysis of matrine against osteosarcoma. (A) Venn diagram showing the overlapping targets of matrine and osteosarcoma. (B) Protein-protein interaction (PPI) network of the core targets.

Figure S5. PPI network of the core targets.

Figure S6. Gene Ontology (GO) and KEGG pathway enrichment analyses (A,B).

Figure S7. Quantification of Western blot analysis in the rescue experiment. Statistical analysis of the relative expression of Mfn1, Mfn2, FIS1, Bax, and cytochrome c in the rescue experiment with 740 Y-P. Data are presented as the mean ± SD.

Table S1 Antibody Information Table

Antibody name	Supplier	Catalog number	dilution ratio
MFN2	Proteintech	67487-1-Ig	1:5,000
MFN1	Diagbio	DB11711	1:1,000
FIS1	Proteintech	66635-1-Ig	1:5,000
Bax	Proteintech	50599-2-Ig	1:2,000
Cytochrome c	Diagbio	DB14687	1:2,000
Phospho-Akt (Ser473)	CST	#4060	1:2,000
Phospho-PI3K (Tyr458)	CST	#17366	1:1,000
β-Actin	Proteintech	60008-1-Ig	1:5,000

Abbreviations: CST, Cell Signaling Technology; MFN2, Mitofusin 2; MFN1, Mitofusin 1; FIS1, Mitochondrial fission 1 protein; Bax, Bcl-2-associated X protein.
