# Supplementary material for: Matrine Suppresses Osteosarcoma Lung Metastasis by Targeting the PI3K/AKT Signaling Pathway to Induce Mitochondrial Dysfunction
Source: Cancers (Basel). 2026 Oct 19;18(20):3381. doi: 10.3390/cancers18203381 (PMC13702236; doi:10.3390/cancers18203381)
Supplement: Supplementary file 1 [file cancers-18-03381-s001.zip › File S1.pdf]

Figure 4

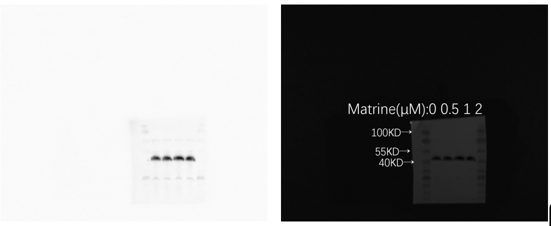

β-actin

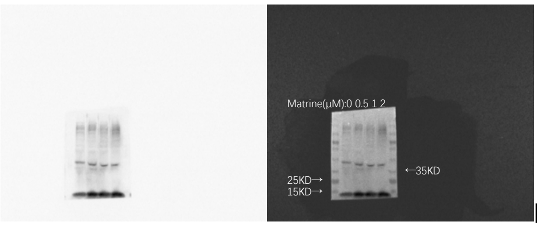

Fis1

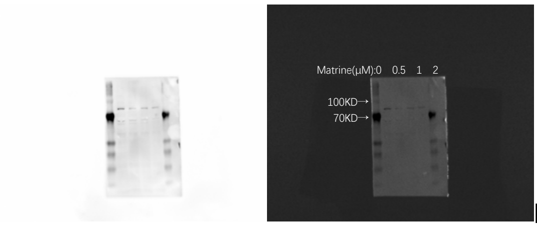

Mfn1

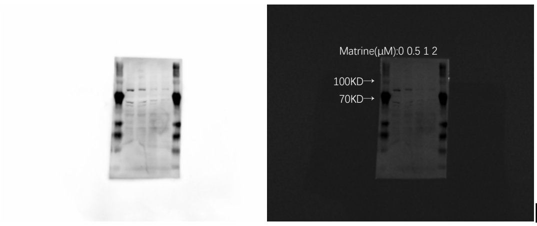

Mfn2

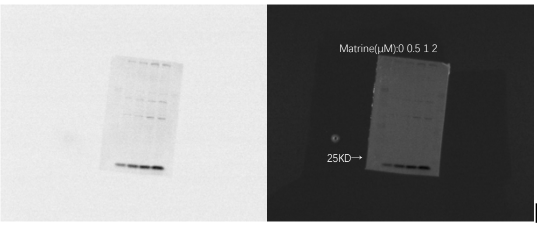

Bax

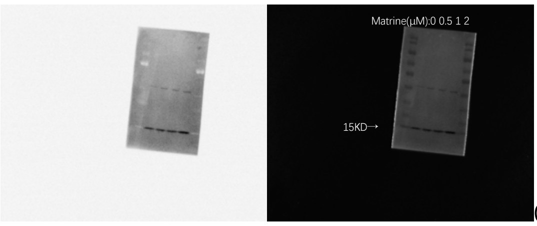

Cyto C

Figure 5

D

DMSO

Actin

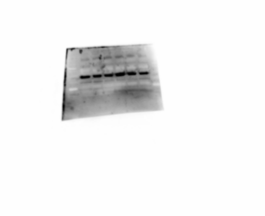

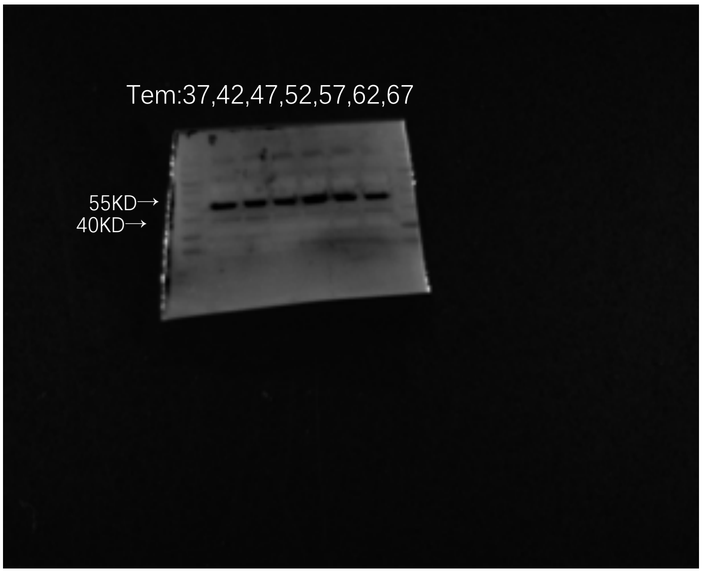

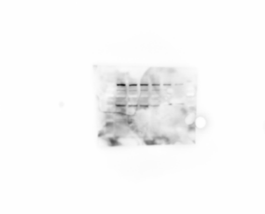

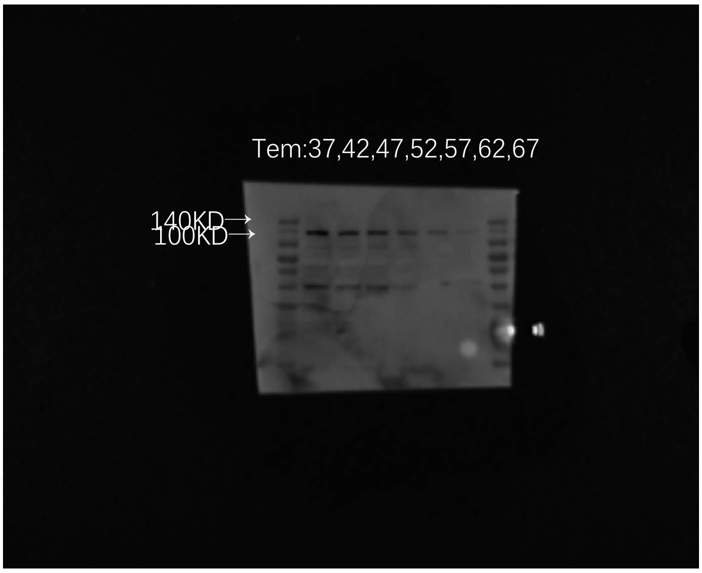

Matrin

β-actin

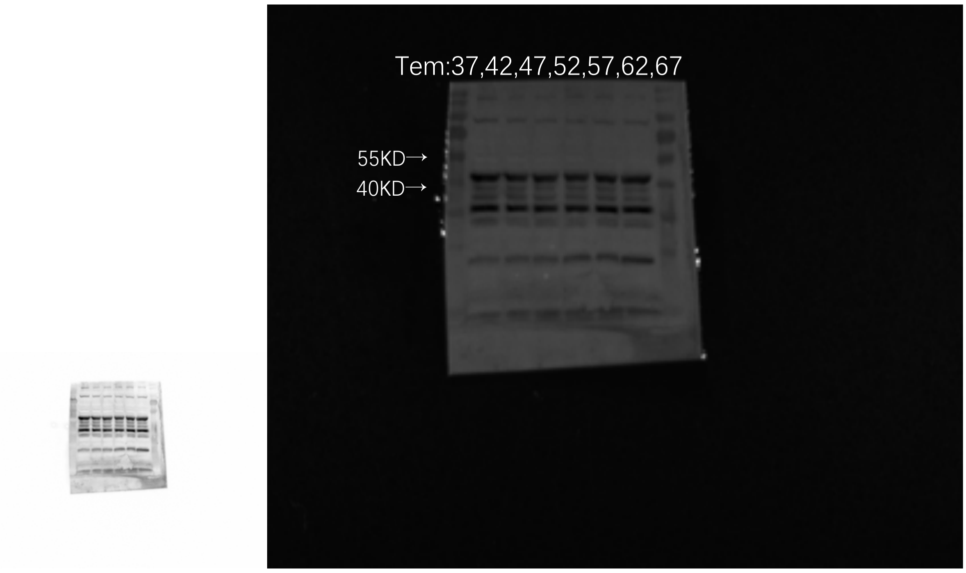
Tem:37,42,47,52,57,62,67
55KD→
40KD→

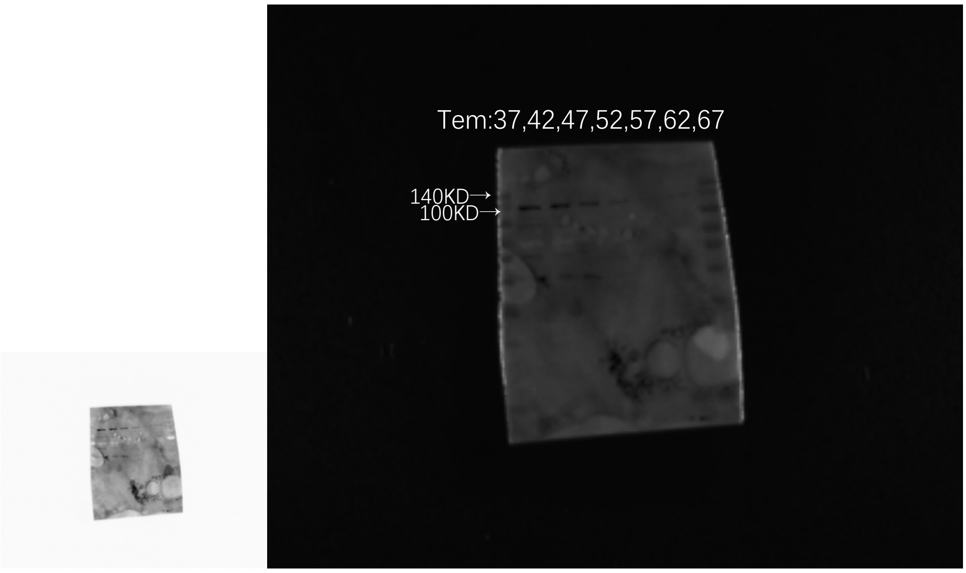
Tem:37,42,47,52,57,62,67
140KD→
100KD→

F

β-actin

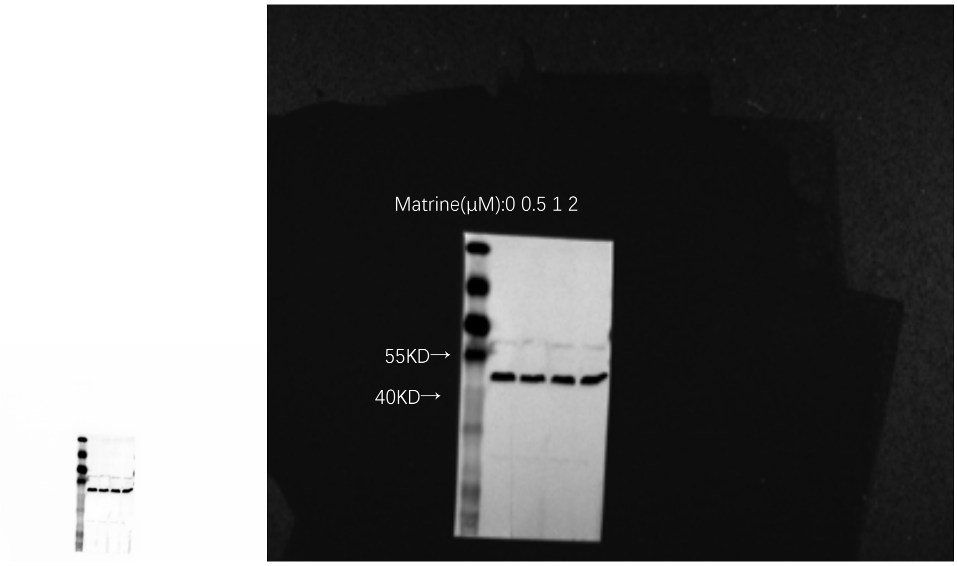
Matrine(μM):0 0.5 1 2
55KD→
40KD→

AKT

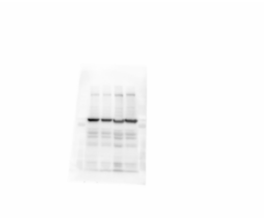

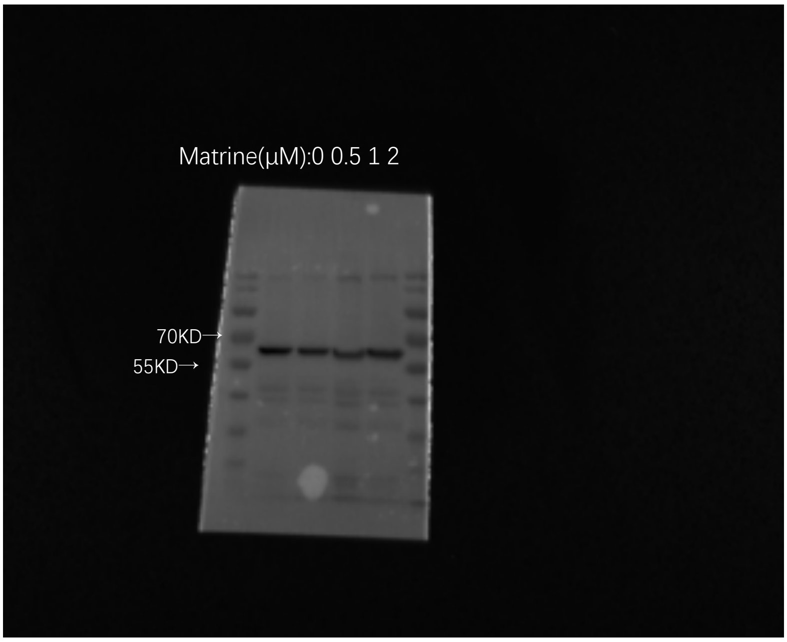

p-AKT

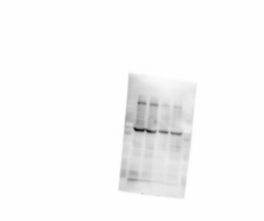

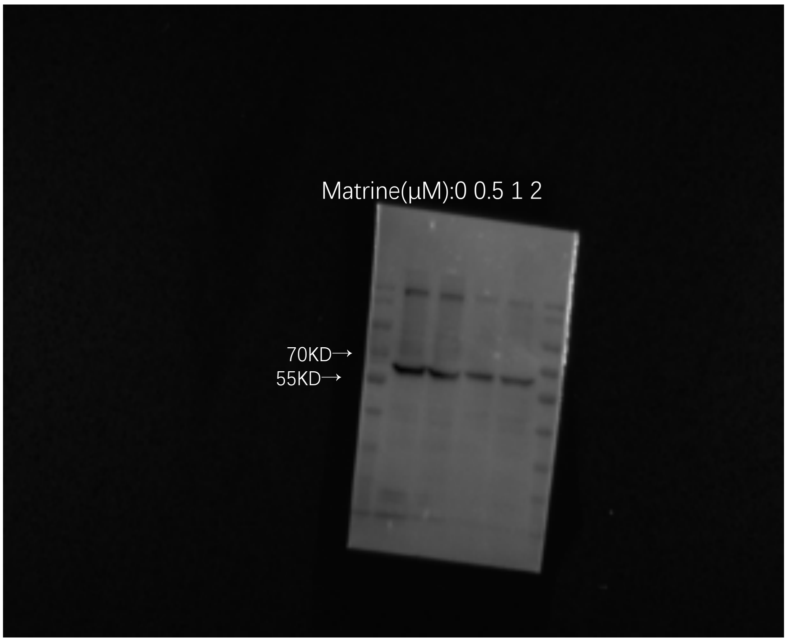

p-PI3K

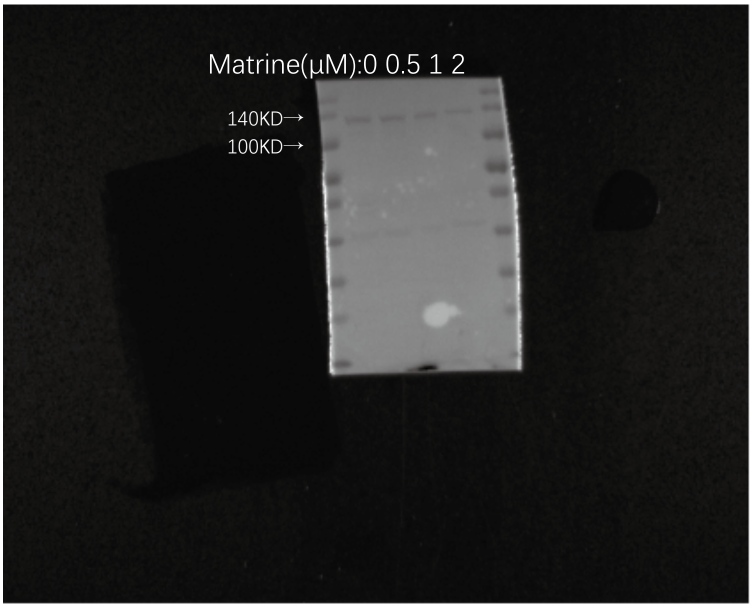

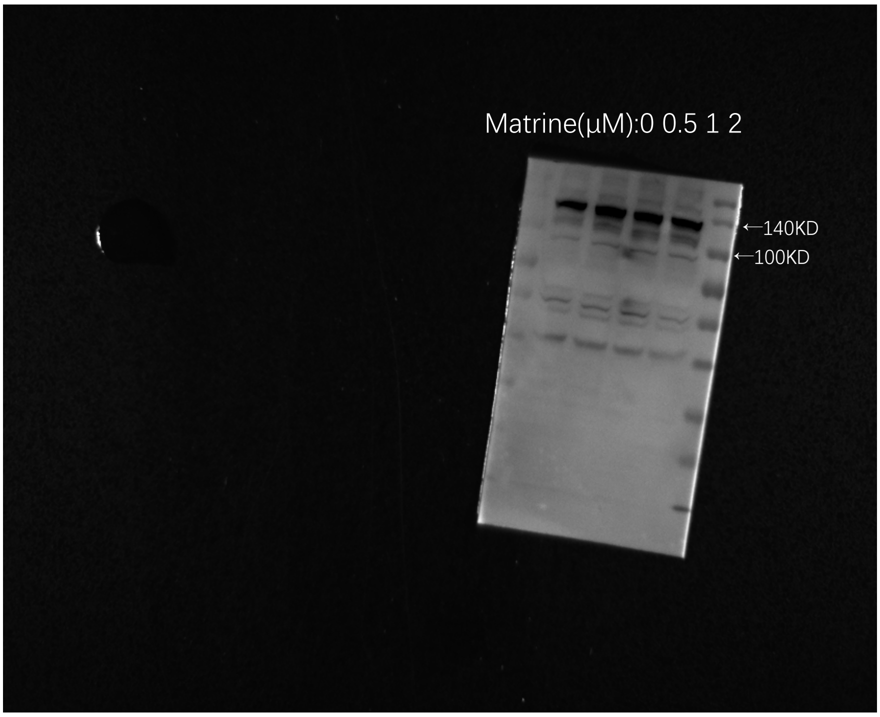

Figure 6

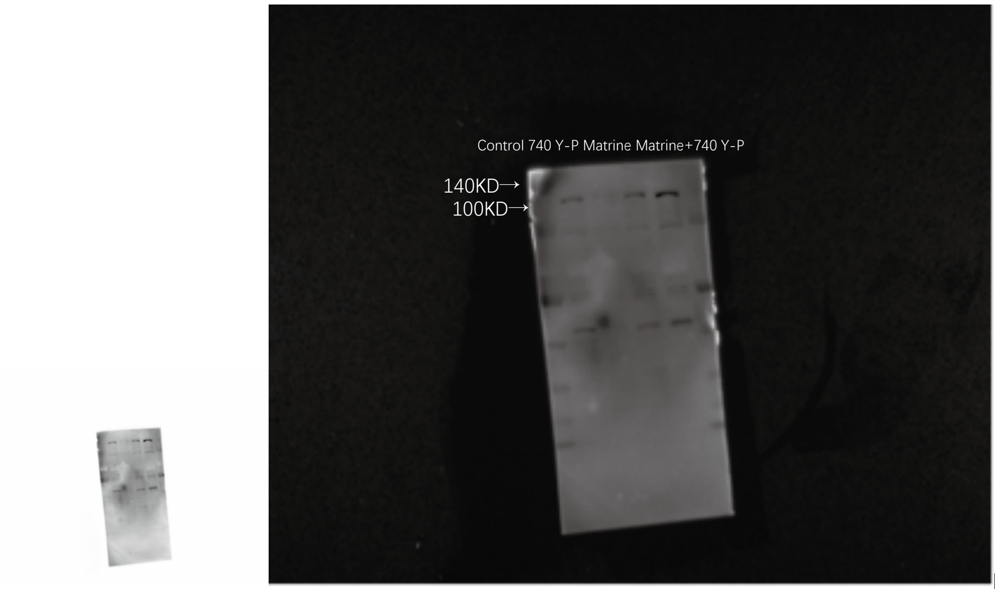

p-PI3K

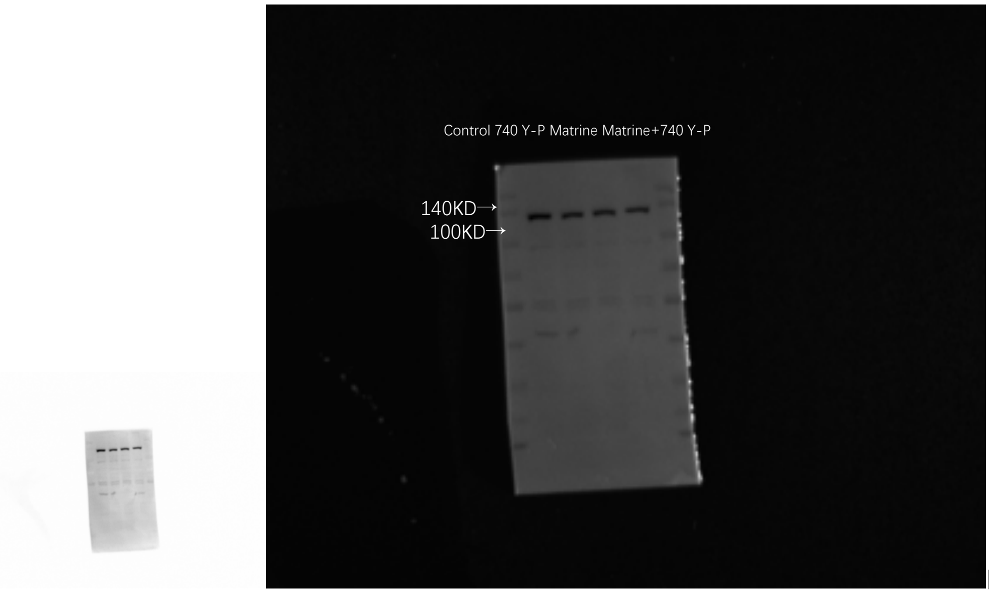

PI3K

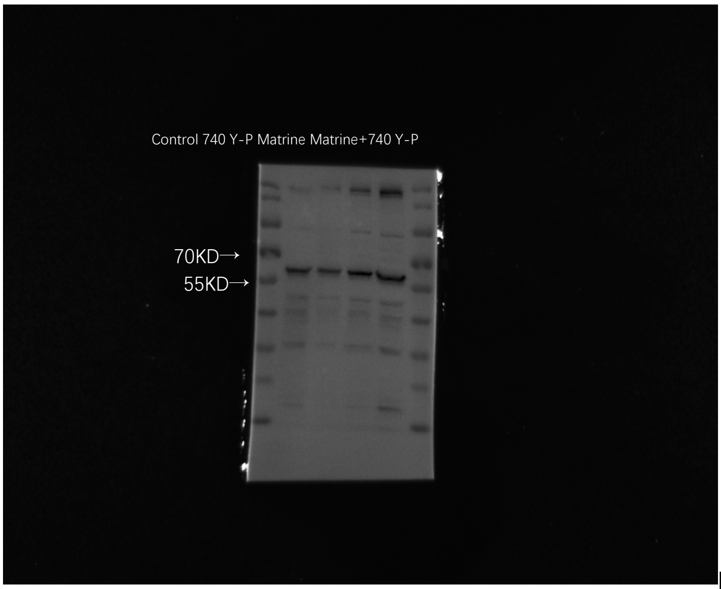

p-AKT

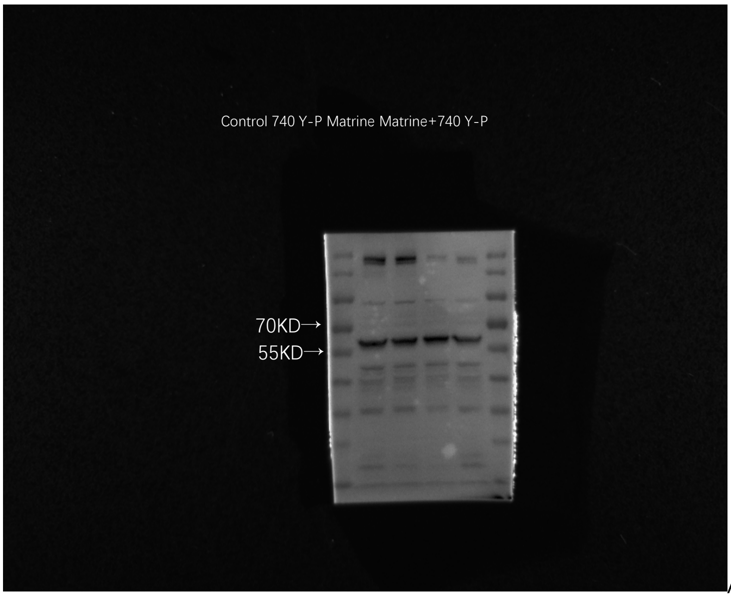

AKT

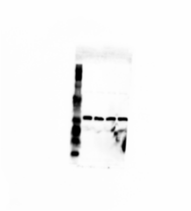

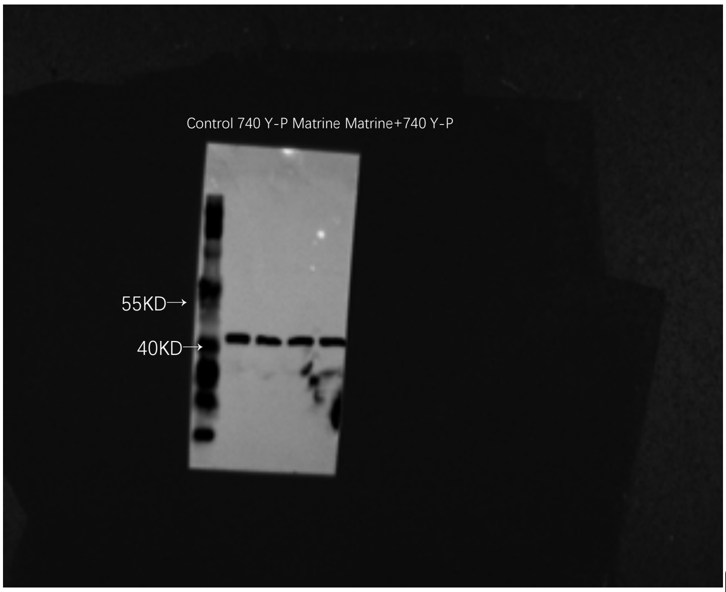

β-actin

Figure 7

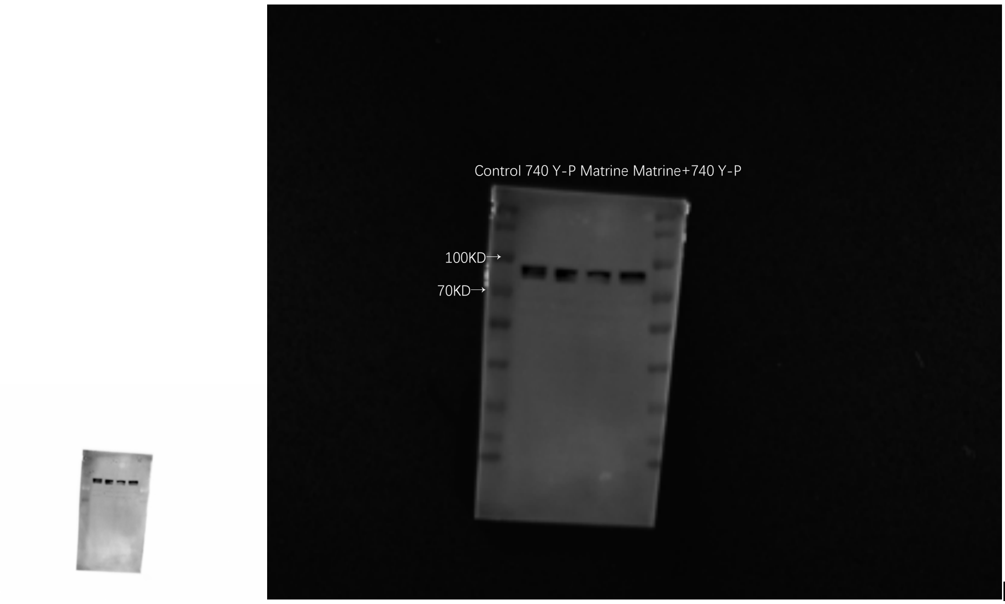

Mfn2

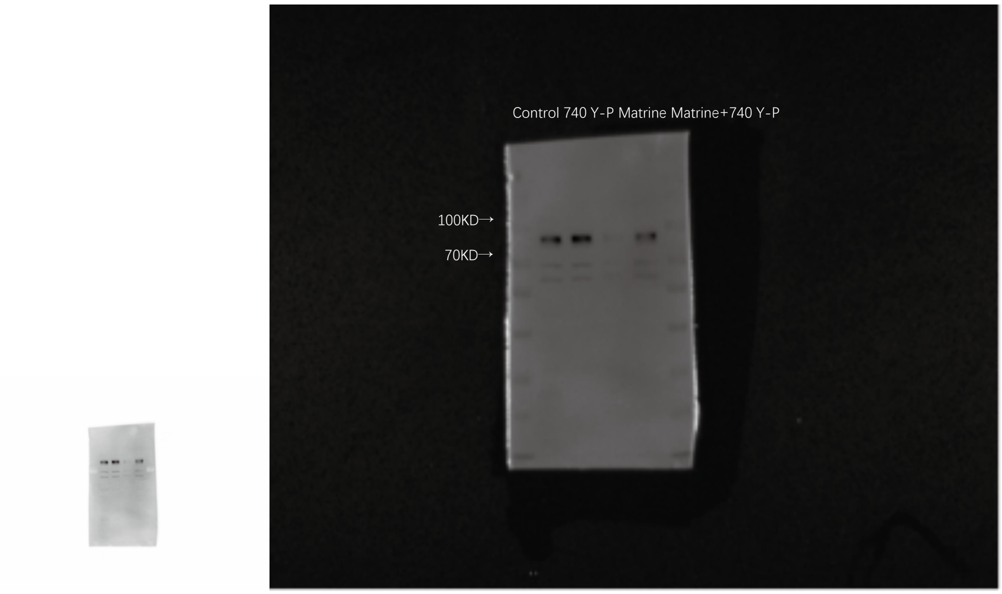

Mfn1

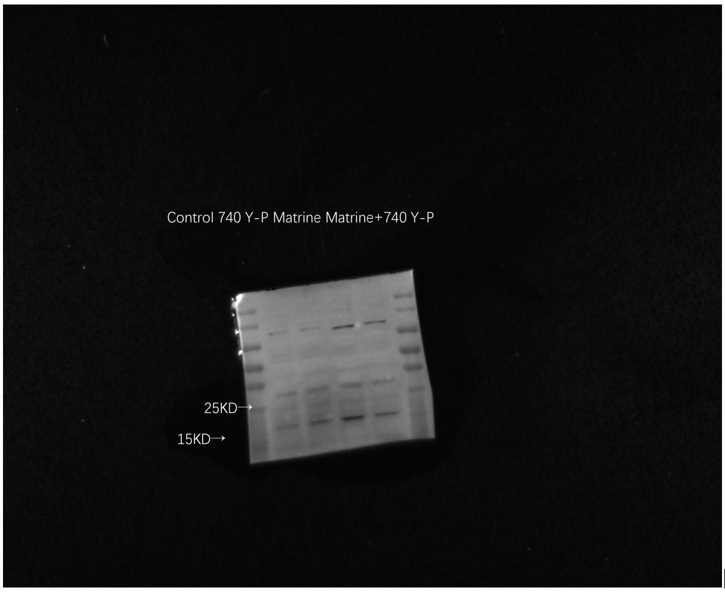

Fis1

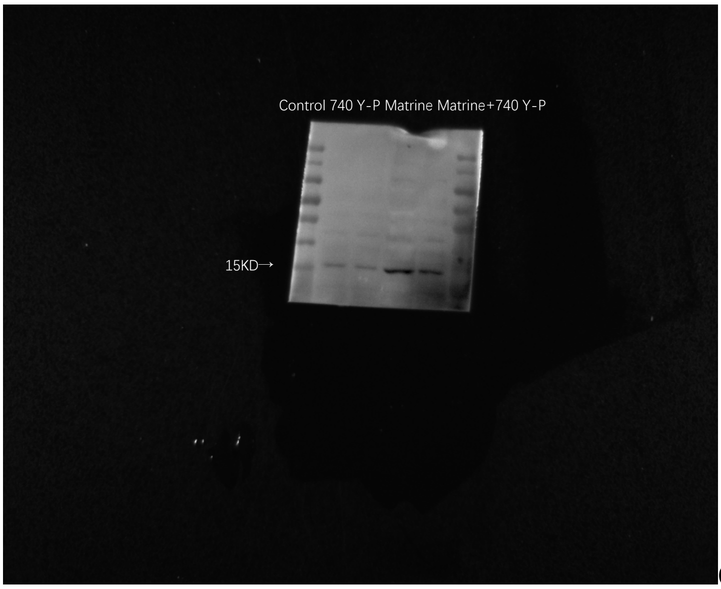

Cyto C

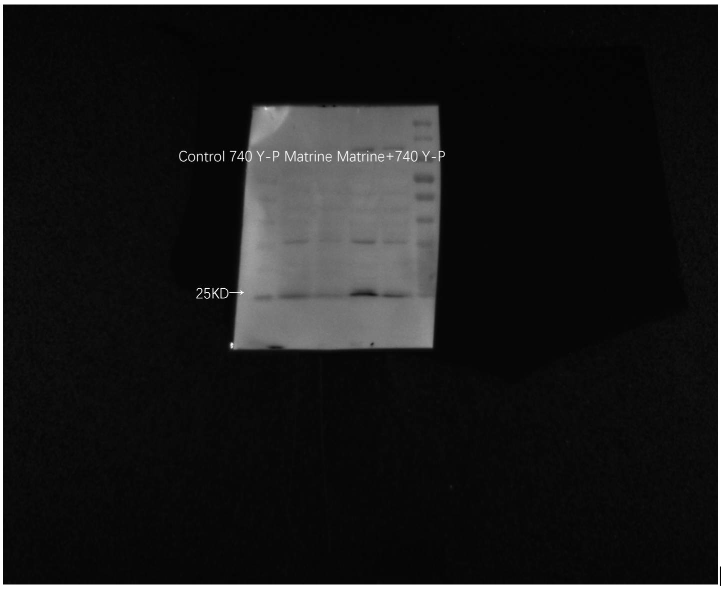

Bax

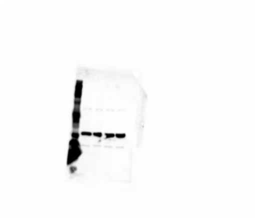

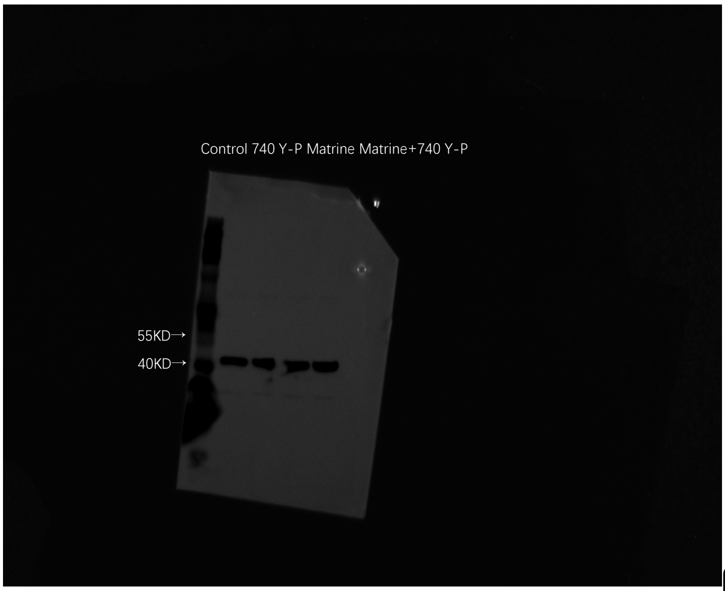

β-actin
